# Genome-Wide and Species-Wide *In Silico* Screening for Intragenic MicroRNAs in Human, Mouse and Chicken

**DOI:** 10.1371/journal.pone.0065165

**Published:** 2013-06-06

**Authors:** Irena Godnic, Minja Zorc, Dasa Jevsinek Skok, George Adrian Calin, Simon Horvat, Peter Dovc, Milena Kovac, Tanja Kunej

**Affiliations:** 1 Department of Animal Science, Biotechnical Faculty, University of Ljubljana, Domzale, Slovenia; 2 Department of Experimental Therapeutics and The Center for RNA Interference and Non-Coding RNAs, The University of Texas, M. D. Anderson Cancer Center, Houston, Texas, United States of America; 3 National Institute of Chemistry, Ljubljana, Slovenia; Philipps University, Germany

## Abstract

MicroRNAs (miRNAs) are non-coding RNAs (ncRNAs) involved in regulation of gene expression. Intragenic miRNAs, especially those exhibiting a high degree of evolutionary conservation, have been shown to be coordinately regulated and/or expressed with their host genes, either with synergistic or antagonistic correlation patterns. However, the degree of cross-species conservation of miRNA/host gene co-location is not known and co-expression information is incomplete and fragmented among several studies. Using the genomic resources (miRBase and Ensembl) we performed a genome-wide *in silico* screening (GWISS) for miRNA/host gene pairs in three well-annotated vertebrate species: human, mouse, and chicken. Approximately half of currently annotated miRNA genes resided within host genes: 53.0% (849/1,600) in human, 48.8% (418/855) in mouse, and 42.0% (210/499) in chicken, which we present in a central publicly available Catalog of intragenic miRNAs (http://www.integratomics-time.com/miR-host/catalog). The miRNA genes resided within either protein-coding or ncRNA genes, which include long intergenic ncRNAs (lincRNAs) and small nucleolar RNAs (snoRNAs). Twenty-seven miRNA genes were found to be located within the same host genes in all three species and the data integration from literature and databases showed that most (26/27) have been found to be co-expressed. Particularly interesting are miRNA genes located within genes encoding for miRNA silencing machinery (*DGCR8*, *DICER1*, and *SND1* in human and *Cnot3*, *Gdcr8*, *Eif4e*, *Tnrc6b*, and *Xpo5* in mouse). We furthermore discuss a potential for phenotype misattribution of miRNA host gene polymorphism or gene modification studies due to possible collateral effects on miRNAs hosted within them. In conclusion, the catalog of intragenic miRNAs and identified 27 miRNA/host gene pairs with cross-species conserved co-location, co-expression, and potential co-regulation, provide excellent candidates for further functional annotation of intragenic miRNAs in health and disease.

## Introduction

MicroRNAs (miRNAs) are non-coding RNAs (ncRNAs) that post-transcriptionally regulate gene expression. The standard dogma states that expression of protein-coding genes is repressed by binding the target gene's complementary sequence in the 3′ untranslated region (3′-UTR) with the miRNA’s seed region: 2–7 or 2–8 consecutive nucleotides from the 5′-end of the miRNA, which are crucial for target recognition [1], [2]. This earlier postulated dogma has now been extended with new discoveries. MicroRNAs have also been shown to increase or decrease expression of protein-coding genes by targeting different genomic regions (3′-UTR, 5′-UTR, promoter, and coding sequences) and interact with proteins. Additionally, they have been shown to function in various subcellular compartments, and developmental and metabolic processes [3]. Several components of the miRNA processing machinery are included in miRNA biogenesis, which first take place in the nucleus. Primary miRNA transcripts (pri-miRNAs) are processed by the complex Drosha-DGCR8 (DiGeorge syndrome critical region gene-8), a component of the miRNA processing machinery [4], [5]. Thereafter precursor miRNAs (pre-miRNAs) are transported to the cytoplasm where they are further cleaved by RNase III Dicer, a key enzyme in miRNA maturation, to form functional mature miRNAs [6]. They are incorporated into the RNA-induced silencing complex (RISC) composed of many associated proteins [7]. Disruption of the miRNA processing machinery core components, miRNA genes and their targets affects overall efficiency of silencing [8]. Indeed, polymorphisms as well as aberrant miRNA expression patterns have previously been shown to be involved in disease development, including several cancer types [9]–[12].

Approximately half of vertebrate miRNAs are processed from introns of protein-coding genes or genes encoding for other ncRNA classes (*e.g.* snoRNAs, miRNAs, lincRNAs) [13], whereas miRNA genes can also be encoded in intergenic regions of DNA, therefore referred to as intergenic miRNAs. In some cases, a miRNA gene can have a “mixed” location, *i*.*e.* can be located either in an exon or an intron of the same or different host gene transcripts which depends on their alternative splicing [13].

A single host gene can comprise multiple and overlapping resident miRNA genes, called a cluster, which are processed from the same polycistronic primary transcript [13], [14]. It has been observed that miRNA genes which are located in a polycistron and co-expressed in the clusters are pivotal in coordinately regulating multiple processes, including embryonic development, cell cycle and cell differentiation [15]. It was also observed that miRNA genes are more frequently hosted within the short genes than expected by chance, which was hypothesized as a favorable evolutionary feature due to the gene’s interaction with the pre-miRNA splicing mechanism [16].

Host genes and resident ncRNAs have been considered to have a synergistic effect with important implications for fine-tuning gene expression patterns in the genome [17], [18]. Expression profiles of intronic miRNAs were in many cases found to coincide with the transcription of their host genes, which raised a question as to how these miRNAs were processed [19]. Intronic miRNAs, like most ncRNAs, are released from the excised host introns in the post-splicing process [17], [20]. However, it was later indicated that intronic miRNAs might also be processed from unspliced intronic regions prior to splicing catalysis [20]. A class of miRNA precursors, named mirtrons, are processed in an alternative miRNA biogenesis pathway where certain debranched introns mimic the structural features of pre-miRNAs and enter the miRNA-processing pathway, however without the Drosha-mediated cleavage [21].

Highly correlated expression patterns have been found in closely clustered miRNA genes (50 kb of each other), which coincides with the idea of a polycistronic primary transcript [19], [22]. He et al. [23] additionally showed that evolutionary conserved miRNA genes tend to be co-expressed with their host genes: even though the non-conserved miRNAs dominate in the human genome, the majority of intragenic miRNAs exhibiting co-expression with their host gene are phylogenetically old. A high conservation between orthologous intronic miRNAs has been demonstrated in several species [24], [25]. In addition to co-expression and proposed co-regulation of miRNA and host genes, several studies have described a functional link between them [19], [26], [27]. Interestingly, genes highly correlated in expression with a resident miRNA gene were found to be more likely predicted as miRNA targets [28]. The expression of miRNA/host genes and that of predicted miRNA targets tend to be positively or negatively correlated, suggesting that the coordinated transcriptional regulation of a miRNA and its target is an abundant motif in gene networks [28].

The proportion of miRNA genes located within the same host genes among different species remains unknown, whether their coordinated expression is conserved, and to what degree. The miRNA/host gene co-expression has been analyzed in several studies, yet the data remains fragmented and incomplete. However, based on the report by He *et al*. [23] that evolutionary conserved (“old”) miRNA genes tend to be co-expressed with their host genes, but, in contrast, non-conserved (“young”) ones rarely do so, it might be reasonable to predict the same co-expression patterns of miRNA/host gene pairs with conserved cross-species co-location. The conserved pairs would present candidate genes whose matching expression profiles would be of assistance for further annotation and functional analysis.

The aim of this study was to create a central Catalog of intragenic miRNAs in three well-annotated vertebrate species (human, mouse, and chicken) serving as a framework for researchers working in the field of intragenic miRNAs. The supplemented information regarding the miRNA/host gene pair’s conserved cross-species co-location, expression data, and disease associations provides a list of high priority intragenic miRNAs for further functional analyses. These include identification and annotation of genes based on cross-species conservation, functional analyses and studies to re-examine potential misattribution of phenotype previously ascribed to host genes or hosted miRNA genes only.

## Materials and Methods

Datasets of miRNA/host gene pairs were downloaded from genomic resources: the coordinates of miRNA genes and their host genes in human, mouse, and chicken were downloaded from miRBase, release 19 (http://www.mirbase.org/) [29] and Ensembl, release 69 (http://www.ensembl.org/index.html), using the latest matching assemblies: GRCh37 for human, GRCm38 for mouse, and WASHUC2 for chicken. The catalog is accessible through a web application written in PHP language, which allows retrieving miRNA/host gene pairs (http://www.integratomics-time.com/miR-host/catalog). The nomenclature of miRNA and host genes was unified according to The HUGO Gene Nomenclature Committee (HGNC) (http://www.genenames.org/) and Mouse Genome Informatics (MGI) (http://www.informatics.jax.org/). The list of miRNA host genes was manually inspected; cases with doubtful gene nomenclature after automatic annotation (*e*.*g*. overwriting of a miRNA record with an overlapping snoRNA and lincRNA record) were reported to the source database (Ensembl) and solved case by case. Genomic distribution of miRNA/host gene pairs in human, mouse, and chicken was presented in a genomic view format using Flash GViewer web tool (http://gmod.org/wiki/Flashgviewer/). MicroRNA and host gene expression profiles, their functional links and diseases associated with dysregulated expression were retrieved from: 1) literature using PubMed (http://www.ncbi.nlm.nih.gov/pubmed), Web of Science (http://apps.webofknowledge.com/), and 2) databases Gene Expression Atlas (GEA), release 2.0.11.1 (http://www.ebi.ac.uk/gxa/). Small RNA expression data was obtained from University of California Santa Cruz (UCSC) Genome Bioinformatics (http://genome.ucsc.edu/) based on the ENCODE project [30]. Genetic variability of miRNA genes residing within host genes (protein-coding and non-coding) was determined using miRNA SNiPer tool 3.0 (http://www.integratomics-time.com/miRNA-SNiPer) [31]. Predicted and experimentally validated miRNA targets were obtained using TargetScan (http://www.targetscan.org/), miRecords (http://mirecords.biolead.org/), and miRTarBase (http://mirtarbase.mbc.nctu.edu.tw/). The list of components of the miRNA silencing machinery was obtained from Patrocles database (http://www.patrocles.org) [32]. Pathway enrichment analysis for miRNA host genes was performed using the Ingenuity Pathway Analysis (IPA), release 8.8 (Ingenuity® Systems, http://www.ingenuity.com/) [33]. Multispecies sequence alignments were performed using Ensembl, option Comparative genomics - Alignments (text).

## Results and Discussion

**Figure 1:**
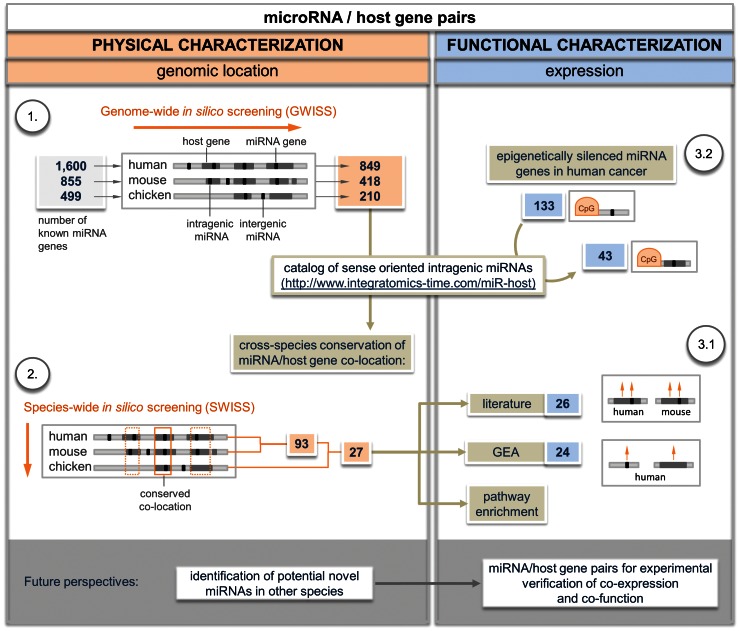
Workflow of the study. GEA – Gene Expression Atlas.

**Figure 2:**
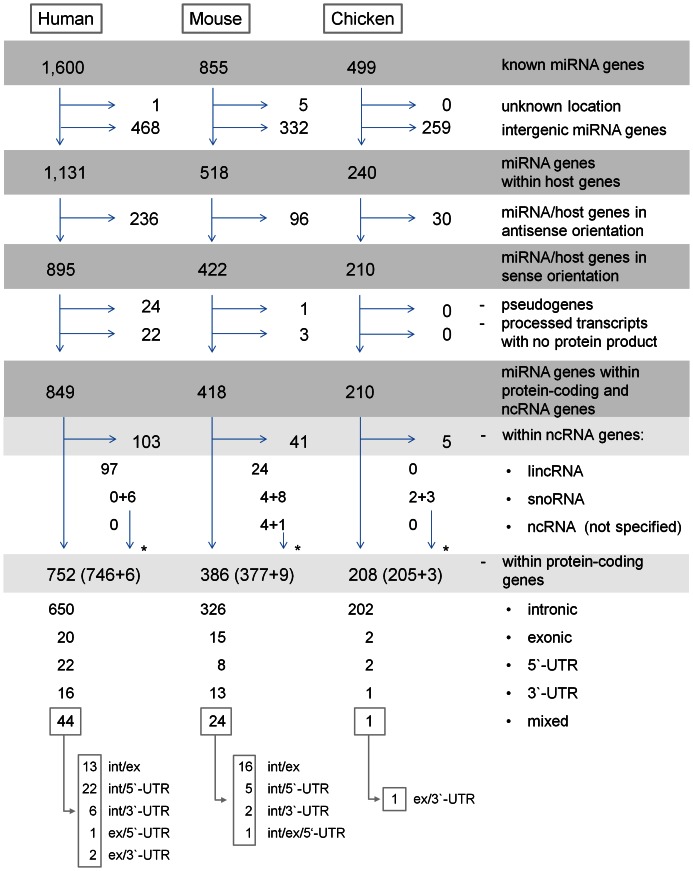
Diagram of genomic distribution of miRNA genes in human, mouse, and chicken. * - microRNA genes overlapping protein-coding and ncRNA genes; mixed - microRNA genes overlapping intron, exon or UTR, depending on overlapping host gene transcripts. For details see online table: http://www.integratomics-time.com/miR-host/catalog.

We developed a central Catalog of intragenic miRNAs in three well-annotated vertebrate genomes (human, mouse, and chicken) by performing a genome-wide *in silico* screening (GWISS) of genomic resource databases (**Figures 1** and **2**). The miRNAs were hosted by protein-coding genes or genes encoding for other ncRNA classes. Further species-wide *in silico* screening (SWISS) revealed 27 miRNA/host gene pairs with conserved co-location in all three species, most of which have been found to be co-expressed. Coordinately expressed miRNA/host gene pairs with cross-species conserved co-location are considered prioritized candidate genes for future functional analysis.

### 1. Genome-wide *in silico* Screening (GWISS) for Sense-oriented miRNA/host Gene Pairs in Human, Mouse and Chicken

**Figure 3:**
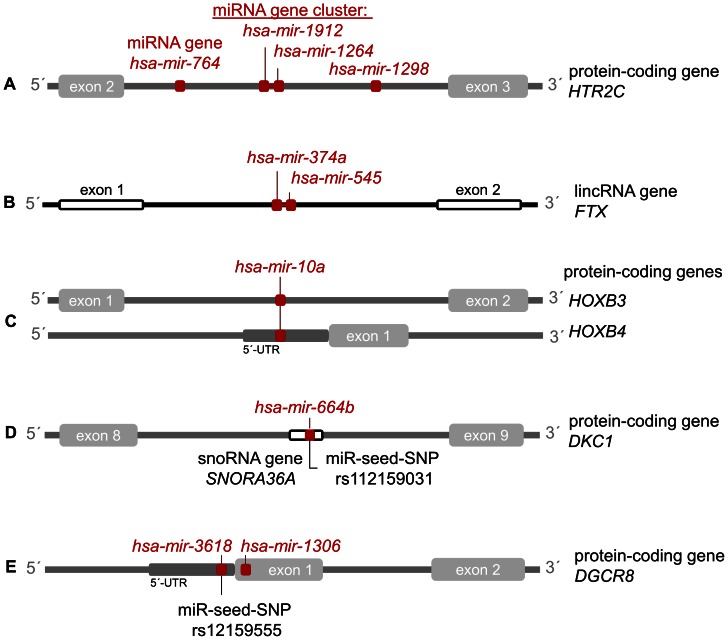
Examples of co-location of miRNA genes with protein-coding and ncRNA genes. **A**) Protein-coding gene *HTR2C* with four resident miRNA genes, two of which form a cluster. **B**) A miRNA gene cluster located within lincRNA gene *FTX*. **C**) MicroRNA gene *hsa-mir-10a* located within two overlapping protein-coding genes. **D**) Overlapping miRNA gene (*hsa-mir-664b*) comprising a miR-seed-SNP, and snoRNA gene (*SNORA36A*) residing within protein-coding *DKC1*. **E**) Gene *DGCR8*, associated with miRNA biogenesis, hosts two miRNA genes, one of which comprises a miR-seed-SNP.

Intragenic miRNAs (**Figure 3**) have become a topic of increasing research interest. We performed a genome-wide *in silico* screening (GWISS) of the latest genome assemblies of three well-annotated vertebrate genomes (human, mouse, and chicken) to define how many miRNA genes are located within host genes. The Catalog of intragenic miRNAs is available through a web application (http://www.integratomics-time.com/miR-host/catalog), which allows users to retrieve single or multiple miRNA/host gene pairs, based on 1) selection of species, biotype of host genes, and genomic position of resident miRNAs (exon, intron, 3′ and 5′-UTR), or 2) by querying individual miRNA or their host genes. In all three species approximately half of currently annotated miRNAs are intragenic, residing within protein-coding and/or ncRNA genes: 53.0% (849/1,600) in human, 48.8% (418/855) in mouse, and 42.0% (210/499) in chicken (**Figure 2**). This percentage however should be considered as an estimate that will change with time as both miRNA and host genes (protein-coding and ncRNA genes) are still being annotated and added to database upgrades. Manual inspection of host genes revealed examples with doubtful annotation in regions with two or three overlapping genes, for which we contacted the source database (Ensembl) and solved ambiguous annotations case by case. Namely, it was observed that in cases where two ncRNA genes (miRNA and snoRNA) overlapped in the same region, the automatic annotation pipeline favored the longer RNA; for example, the record of snoRNA gene *SNORA36B* overwrote the record of the overlapping miRNA gene *hsa-mir-664a*. One of the reasons for annotation error may also be the use of non-official and inconsistent nomenclature of genes. For example, a miRNA host transcript with a lincRNA biotype (ENSG00000253522) was merged between the Ensembl automatic pipeline and the Havana manual curation and was found to be given two names, *CTC-231O11.1* or *hsa-mir-146a*. Any updates of the catalog of miRNA/host gene pairs should therefore take into consideration the importance of nomenclature when searching for single or overlapping miRNA genes.

MicroRNA genes that do not share the same strand orientation as their host genes (*i.e.* are antisense-oriented) have been shown to have independent transcription mechanisms [34], whereas sense transcriptional orientation suggests that miRNA and host genes can be transcribed from shared promoters [1]. Additionally, it was found that a majority of predicted promoter regions of intronic miRNA genes (94.2%; 49/52) overlapped with their host gene promoters [35]. In addition to protein-coding host genes, ncRNA genes comprised snoRNAs, lincRNAs, and other unspecified ncRNAs (**Figure 2**). Long ncRNAs were found to also host clusters of miRNA genes and therefore encode polycistronic primary transcripts that can yield several miRNAs; for example lincRNA *FTX* (FTX transcript, XIST regulator (non-protein coding)) comprises two miRNA genes: *hsa-mir-374a* and *hsa-mir-545* (**Figure 3B**). Because miRNA clusters can also overlap with a single protein-coding host gene (**Figure 3A**), the total number of host genes is lower than the number of intragenic miRNAs: we identified 687 protein-coding host genes in human (with 752 resident miRNA genes), 288 in mouse (with 386 miRNA genes), and 192 in chicken (with 208 miRNA genes). In all three species intragenic miRNA clusters most frequently comprise two miRNAs per host gene, as shown in the online table: http://www.integratomics-time.com/miR-host/catalog. The mouse host gene *Sfmbt2* (Scm-like with four mbt domains 2), located on MMU2, was found to comprise the largest number of resident miRNA genes (n = 70) belonging to the *mir-297*, *mir-466*, and *mir-467* gene families. Our study revealed that around one tenth of miRNA genes formed clusters in protein-coding host genes: 8.8% (141/1,600) in human, 14.5% (124/855) in mouse, and 8.2% (41/499) in chicken. It was also proposed that human miRNAs that share a host gene or are organized in clusters might also, due to clustering propensity, share a significant biological role [36], [37]. Accordingly, miRNA genes that formed clusters were also found to be coordinately expressed with their host genes, which will be described in section 3.

For all three species (human, mouse, and chicken) we presented online genomic-views of intragenic miRNAs genes, connected to miRBase and host genes connected to Ensembl, with an outgoing link (http://www.integratomics-time.com/miR-host/GViews). The human genomic-view is presented in **Figure S1**. Intragenic miRNAs were found distributed among all chromosomes, however some, *e.g*. HSA14, HSA19, and HSAX, were found to comprise less intragenic miRNA genes compared to other chromosomes (**Figure S2**). In most cases miRNA genes resided within a single host gene. For example, human *hsa-mir-1307* gene overlaps with a single host gene *USMG5* (up-regulated during skeletal muscle growth 5 homolog (mouse)) gene. On the other hand, ten miRNA genes were found to overlap with two protein-coding host genes in human (http://www.integratomics-time.com/miR-host/human_coding). For example *hsa-mir-10a* overlapped with both, *HOXB3* (homeobox B3) and *HOXB4* (homeobox B4) (**Figure 3C**). Regarding the location of miRNA genes, we found that in accordance with previous publications [13], [20], [38] a majority of intragenic miRNA genes were located within introns of their protein-coding host genes: 86.4% (650/752) in human, 84.4% (326/386) in mouse, and 97.1% (202/208) in chicken (**Figure 2**). Intronic miRNAs were also most frequently found to be coordinately expressed with their host genes among species, which will be further discussed in results section 2 and 3.

#### 1.1. Co-location of miRNA with other ncRNA genes

Besides the half of miRNAs located within protein-coding genes, we found that around 4% were positioned within genes encoding for other ncRNA classes. These include lincRNAs, snoRNAs, or other ncRNAs: 6.4% (103/1,600) in human, 4.8% (41/855) in mouse, and 1% (5/499) in chicken, which can be accessed at http://www.integratomics-time.com/miR-host/catalog. Nomenclature conflicts of miRNA and ncRNA names may occur due to annotation difficulties: information merged from the Ensembl automatic pipeline and the Havana manual curation, which assign gene names according to miRBase and the HUGO Gene Nomenclature Committee. Six human miRNA genes were found located in both, protein-coding and ncRNA genes: *hsa-mir-600*, *-664a*, *-664b*, *-1248*, *-1291*, and *-3651* (**online table** http://www.integratomics-time.com/miR-host/human_table). MicroRNA gene *hsa-mir-664b*, its overlapping protein-coding host gene *DKC1* (dyskeratosis congenita 1, dyskerin) and snoRNA *SNORA36A* gene are shown in **Figure 3D**. Some miRNA genes were found to form clusters within hosting ncRNA genes: for example the miRNA gene cluster, comprising *hsa-mir-374a* and *hsa-mir-545*, is located within lincRNA gene *FTX* (**Figure 3B**). Additionally, lincRNAs have also been found to be the most frequent type of ncRNA host genes (97/103) as shown in the online table: http://www.integratomics-time.com/miR-host/human_table. In some cases the designated lincRNAs have been found to be the primary transcripts and not actual lincRNA genes, for example *MIR155HG* (also known as *BIC*) and *DLEU2* (deleted in lymphocytic leukemia 2 (non-protein coding), previously known as *LEU2*, are primary transcripts of their resident miRNA genes *hsa-mir-155* and *hsa-mir-15a/16-1*, respectively. Besides miRNAs themselves being regulators of gene expression participating in a wide regulatory network [1], [3], their long ncRNA genes have likewise been found associated with human diseases. For example, lincRNA *H19* (H19, imprinted maternally expressed transcript (non-protein coding)), which hosts *hsa-mir-675*, was implicated in human tumor growth [39] in esophageal [40] and breast cancer [41], and different carcinomas and hepatic metastases [42]. Another study demonstrated that *H19* and *hsa-mir-675* were upregulated in human colon cancer cell lines and primary colorectal cancer tissues [43]. Long intergenic ncRNA *MEG3* (maternally expressed gene 3) could act as a tumor suppressor [44], while both the miRNA gene *hsa-mir-155* and *BIC* RNA (*MIR155HG*) from which it is processed, were overexpressed in human B-cell lymphomas [45]. Similarly, it was shown that the deletion of the 13q14 region, which encodes both, lincRNA *DLEU2* and its resident miRNA cluster *hsa-mir-15a/16-1*, led to chronic lymphocytic leukemia in both human [46] and mouse [47].

#### 1.2. Genetic variability of intragenic miRNA genes

The intragenic miRNAs were also analyzed for genetic variability within the miRNA seed region (miR-seed-SNPs). By analyzing variation databases we found that 14.2% of intragenic miRNAs had polymorphic seed regions in human (121/849), 2.1% in mouse (9/418), and 1.4% in chicken (3/210) (**Table S1**). According to the NCBI database 18 out of 121 miRNA genes in human and two murine miRNA genes have not yet had validated miRNA seed polymorphisms. The actual proportion of polymorphic miRNA genes cannot yet be determined because miRNAs and polymorphisms, most of which are experimentally unvalidated, are still being discovered and added to the databases. That is why the results from previous studies tend to differ: Saunders *et al.* [48] found that less than 1% (3/474) of human miRNA genes miR-seed-SNPs, whereas in our previous study, Zorc *et al.* [31], we reported that 5.9% of miRNA genes comprised miR-seed-SNPs. Polymorphic miRNA genes are an interesting feature to include in the host gene analysis because they have previously been found to have functional associations. For example, we found a link between two independent studies: human *MYH7B* gene (myosin, heavy chain 7B, cardiac muscle, beta) hosts *hsa-mir-499a*, a miRNA upregulated in human and murine cardiac hypertrophy and cardiomyopathy [49], which comprises miR-seed-SNP rs3746444 linked with increased risk of dilated cardiomyopathy [50]. A similar overlap was demonstrated previously comprising a mouse miRNA gene *mmu-mir-717*, a miR-seed-SNP identified in the lean mouse strain 129/Sv, a body mass associated host gene *Gpc3* (glypican 3), as well as a growth associated quantitative trait locus (QTL) [51]. Our catalog provides the basis for a more targeted selection of SNPs and functional connections with the miRNA and host genes.

#### 1.3. MicroRNA/host gene pairs in miRNA biogenesis and regulation

**Figure 4:**
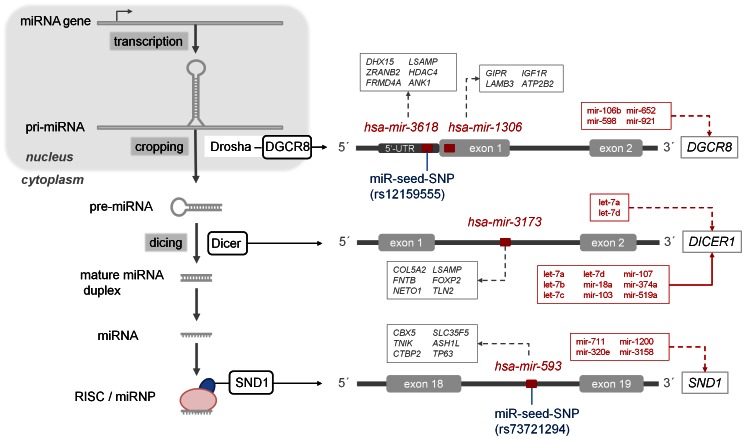
Cross talk of miRNA-related genomic elements. Overlapping miRNA genes (*hsa-mir-3618* and *mir-1306*, *mir-3173*, and *mir-593*), miRNA polymorphisms (miR-seed-SNPs (rs12159555 and rs73721294), host genes encoding for miRNA processing machinery components (*DGCR8*, *DICER1*, and *SND1*), miRNA target sites within host genes, and miRNAs targeting other host genes. Arrow with solid line: experimentally validated miRNA targets; arrow with dashed line: predicted miRNA targets.

By considering the host gene’s function our study revealed an interesting observation that miRNAs are also located within genes encoding for components of the miRNA processing machinery. There were four miRNAs in human located within genes encoding for components of miRNA biogenesis: *DGCR8*, *DICER1*, and *SND1* (**Figure 4**). Similarly, five miRNA genes in mouse were located within *Cnot3*, *Dgcr8*, *Eif4e*, *Tnrc6b*, and *Xpo5* (**Figure S3**). Two miRNA genes (*hsa-mir-1306* and *hsa-mir-3618*) reside within gene *DGCR8*, whose protein product is essential for miRNA biogenesis (**Figure 3E**). Human miRNA gene *hsa-mir-3173*, was found located within an intron of host gene *DICER1*, encoding a protein that functions as a ribonuclease required to produce active RNAs. MicroRNA gene *hsa-mir-593* resided within an intron of *SND1* (staphylococcal nuclease and tudor domain containing 1), a component of RISC. By performing a target gene analysis we found that each of the residing miRNAs was predicted to target genes which also host other miRNA genes (**Figure 4**). According to previous experimental studies, *DICER1* was found targeted by nine miRNAs: *hsa-let-7a*, -*7b*, -*7c*, and *-7d*, *hsa-mir-18a*, *-103*, *-107*, *-374a*, and *-519a* [52]–[55]. Additionally, *hsa-mir-3618* and *hsa-mir-593* were found to comprise a miR-seed-SNPs (rs12159555 and rs73721294, respectively), however both SNPs still need to be validated. Where miRNA molecule targets a gene from a miRNA processing machinery this could indicate a negative regulatory loop and a multi-layer regulatory cross-point, possibly associated with the disrupted processing of miRNAs. Also, alterations in gene regulation could have pathologic implications, as all three miRNA silencing machinery genes have previously been linked to certain diseases: *DICER1* with cancer [11], [56], *DGCR8* with DiGeorge syndrome [57], and *SND1* was found frequently up-regulated in human and mouse cancers, as well as in aberrant crypt foci [58]. To summarize, this miRNA-related genomic cross-points consists of: 1) intragenic miRNAs, 2) miRNA gene polymorphisms, 3) miRNA host genes encoding for proteins involved in miRNA biogenesis and silencing, 4) miRNA target sites within miRNA host genes, and 5) their resident miRNAs targeting other host genes. Polymorphisms and aberrations in this miRNA-related and disease-associated genomic cross-point could therefore have a significant effect on phenotypic variation, including disease susceptibility and deserve further analysis.

### 2. Cross-species Conservation of miRNA/host Gene Co-location

In order to determine how many intragenic miRNAs are located within the same host genes in human, mouse, and chicken, we performed a species-wide *in silico* screening (SWISS) of their co-location. We found that 27 miRNA genes had conserved co-location within the same 23 host genes in all three species (**Table 1**, **Figure S4**). In some cases the host genes (*NFYC*, *SMC4*, and *C9orf3*) encompassed more than one resident miRNA, explaining the co-location of the 27 miRNAs within 23 host genes. Moreover, additional 93 miRNA/host gene pairs were found to have conserved co-location in human and mouse (**online table:** http://www.integratomics-time.com/miR-host/species_cons). Most of the intragenic miRNAs were found to reside within introns of their host genes (25/27) (**Table 1**). MicroRNA/host gene pairs with conserved co-location offer a foundation for structural annotation of novel miRNA genes in other species. Using this approach, we proposed a novel miRNA gene in chicken (*mir-3064*) based on its pre-miRNA region that was found conserved in human and mouse (**Figure S5**). Similarly, 15 potential miRNA genes in human have been suggested by comparing the annotated murine miRNA genes with the human genome. Sequences of potential human miRNAs were examined for small RNA expression data using the UCSC database. Four of the human sequences (complementary to mouse *mmu-mir-677*, -*1839*, -*1897*, and -*1949*) had available expression data (**Figure S5**), which further confirms that these sequences encode miRNAs. The proposed novel miRNA genes present candidates for further experimental validation, annotation and expression analysis. In this manuscript the proposed miRNAs (one in chicken and 15 in human) have been given temporary names and will be submitted to the miRBase upon acceptance of this manuscript by the peer review process.

**Table 1:** Twenty-seven miRNA/host gene pairs with conserved co-location in human, mouse, and chicken.

Human			Mouse			Chicken		
miRNA gene	location within host gene	host gene (synonym)	miRNA gene	location within host gene	host gene (synonym)	miRNA gene	location within host gene	host gene (synonym)
*let-7g*	intron 2, 3	*WDR82*	*let-7g*	intron 2	*Wdr82*	*let-7g*	intron 2	*WDR82_CHICK*
*mir-101-2*	intron 4, 7, 8	*RCL1*	*mir-101b*	intron 8	*Rcl1*	*mir-101*	intron 8	*RCL1*
*mir-103a-1*	intron 2, 5	*PANK3*	*mir-103-1*	intron 5	*Pank3*	*mir-103-1*	intron 4, 5	*PANK3*
*mir-107*	intron 4, 5	*PANK1*	*mir-107*	intron 5	*Pank1*	*mir-107*	intron 5	*PANK1*
*mir-126*	intron 5–7	*EGFL7*	*mir-126*	intron 1, 6, 7, exon 4	*Egfl7*	*mir-126*	intron 7	*EGFL7*
*mir-128-1*	intron 7, 15, 18	*R3HDM1*	*mir-128-1*	intron 19	*R3hdm1*	*mir-128-1*	intron 18	*R3HDM1*
*mir-128-2*	intron 3, 6, 17, 18	*ARPP21*	*mir-128-2*	intron 14–17, 19	*Arpp21*	*mir-128-2*	intron 16	*ARPP21*
*mir-1306*	5′-UTR	*DGCR8*	*mir-1306*	exon 1–3	*Dgcr8*	*mir-1306*	5′-UTR	*DGCR8*
*mir-140*	intron 3, 6, 7, 9, 14, 16	*WWP2*	*mir-140*	intron 16	*Wwp2*	*mir-140*	intron 16, 17	*WWP2*
*mir-15b*	intron 1–5	*SMC4*	*mir-15b*	intron 5	*Smc4*	*mir-15b*	intron 4	*SMC4*
*mir-16-2*	intron 1–5	*SMC4*	*mir-16-2*	intron 5	*Smc4*	*mir-16-2*	intron 4	*SMC4*
*mir-190a*	intron 22, 27, 51, 53	*TLN2*	*mir-190a*	intron 53	*Tln2*	*mir-190*	intron 54	*TLN2*
*mir-211*	intron 4–7	*TRPM1*	*mir-211*	intron 1, 2, 4–6	*Trpm1*	*mir-204-2* (orthologue)	intron 5	*TRPM1*
*mir-218-1*	intron 14–16	*SLIT2*	*mir-218-1*	intron 1, 8, 14–16	*Slit2*	*mir-218-1*	intron 13, 15	*Q90XG3_CHICK* (orthologue)
*mir-218-2*	intron 4, 14	*SLIT3*	*mir-218-2*	intron 14	*Slit3*	*mir-218-2*	intron 1, 9	*Q90Z43_CHICK* (orthologue)
*mir-23b*	intron 4–6, 14, 15	*C9orf3*	*mir-23b*	intron 3, 15	*2010111I01Rik* (orthologue)	*mir-23b*	intron 15	*C9orf3*
*mir-24-1*	intron 4–6, 14,15, exon 7	*C9orf3*	*mir-24-1*	intron 3, 15	*2010111I01Rik* (orthologue)	*mir-24*	3′-UTR	*C9orf3*
*mir-26a-1*	intron 2, 4, 5	*CTDSPL*	*mir-26a-1*	intron 4, 5	*Ctdspl*	*mir-26a*	intron 5	*CTDSL_CHICK*
*mir-27b*	intron 4–6, 14, 15	*C9orf3*	*mir-27b*	intron 3, 15	*2010111I01Rik* (orthologue)	*mir-27b*	intron 15	*C9orf3*
*mir-301a*	intron 1	*SKA2*	*mir-301a*	intron 1	*SKA2 (Fam33a)*	*mir-301b* (orthologue)	intron 1	*SKA2*
*mir-30c-1*	intron 1–6, 10	*NFYC*	*mir-30c-1*	intron 3–5	*Nfyc*	*mir-30c-1*	intron 4	*NFYC*
*mir-30e*	intron 1–6, 10	*NFYC*	*mir-30e*	intron 3–5	*Nfyc*	*mir-30e*	intron 4	*NFYC*
*mir-32*	intron 8, 12, 14	*TMEM245 (C9orf5)*	*mir-32*	intron 8, 12, 14	*TMEM245*	*mir-32*	intron 23	*Tmem245 (C9orf5)*
*mir-33a*	intron 1, 2, 9, 10, 16, 18, 19	*SREBF2*	*mir-33*	intron 16	*Srebf2*	*mir-33 (mir-33-1)*	intron 13	*SREBF2*
*mir-455*	intron 5–7, 10	*COL27A1*	*mir-455*	intron 7, 10	*Col27a1*	*mir-455*	intron 18	*COL27A1*
*mir-499a*	intron 20	*MYH7B*	*mir-499*	intron 19	*Myh7b*	*mir-499*	intron 18, 19	*MYH7B*
*mir-7-1*	intron 1, 3, 15, 16	*HNRNPK*	*mir-7a-1*	intron 2, 3, 5, 7, 11, 14, 15, 17	*Hnrnpk*	*mir-7-1*	intron 15	*HNRNPK*

Host gene names: *ARPP21*: cAMP-regulated phosphoprotein, 21kDa; *COL27A1*: collagen, type XXVII, alpha 1; *CTDSPL*: CTD (carboxy-terminal domain, RNA polymerase II, polypeptide A) small phosphatase-like; *C9orf3*: chromosome 9 open reading frame 3; *C9orf5*: chromosome 9 open reading frame 5; *EGFL7*: EGF-like-domain, multiple 7; *Fam33a*: spindle and kinetochore associated complex subunit 2; *DGCR8*: DiGeorge syndrome critical region gene 8; *HNRNPK*: heterogeneous nuclear ribonucleoprotein K; *MYH7B*: myosin, heavy chain 7B, cardiac muscle, beta; *NFYC*: nuclear transcription factor Y, gamma; *PANK1*: pantothenate kinase 1; *PANK3*: pantothenate kinase 3; *R3HDM1*: R3H domain containing 1; *RCL1*: RNA terminal phosphate cyclase-like 1; *SKA2*: spindle and kinetochore associated complex subunit 2; *SLIT2*: slit homolog 2 (Drosophila); *SLIT3*: slit homolog 3 (Drosophila); *SMC4*: structural maintenance of chromosomes 4; *SREBF2*: sterol regulatory element binding transcription factor 2; *TLN2*: talin 2; *TMEM245*: transmembrane protein 245; *TRPM1*: transient receptor potential cation channel, subfamily M, member 1; *WDR82*: WD repeat domain 82; *WWP2*: WW domain containing E3 ubiquitin protein ligase 2.

### 3. Coordinated Expression and Functional Association of miRNA/host Gene Pairs

To find out whether miRNA/host gene pairs with conserved cross-species co-location are also co-expressed, we integrated experimental data from two different sources: published studies that experimentally confirmed miRNA/host gene co-expression and databases providing gene expression data for miRNA and host genes separately.

#### 3.1. Co-expression of miRNA/host gene pairs with conserved cross-species co-location

For the first step in determining if the 27 miRNA/host gene pairs with conserved cross-species co-location (in human, mouse, and chicken) (**Table 1**) are also co-expressed, we analyzed data from 28 studies that experimentally confirmed their coordinated expression [19], [26], [27], [59]–[71]. The data integration revealed that most miRNA/host gene pairs (26/27) have previously been found to be coordinately expressed (either both up- or down-regulated) in human and/or mouse (**online table**: http://www.integratomics-time.com/miR-host/co-exp). Co-expression of only one miRNA/host gene pair, *mir-1306*/*DGCR8*, has not yet been experimentally demonstrated. We also found opposing results regarding the expression of two miRNA/host gene pairs, murine *mmu-mir-103/Pank3* and *mmu-mir-107/Pank1–* these have previously been demonstrated to have coordinate [71] as well as anti-correlative (or discordant) expression patterns [72]. Out of the 26 miRNA/host gene pairs with coordinated expression, 11 have been found to be coordinately expressed in both, human and mouse [19], [27], [59], [61]–[64], [67]–[69], [71], [73]–[79]: *mir-103*/*PANK3*, *mir-107*/*PANK1*, *mir-126*/*EGFL7*, *mir-128-1*/*R3HDM1*, *mir-140*/*WWP2*, *mir-211*/*TRPM1*, *mir-218-1*/*SLIT2*, *mir-218-2*/*SLIT3*, *mir-27b*/*C9orf3*, *mir-33*/*SREBF2*, and *mir-499*/*MYH7B*. Moreover, two miRNA/host gene pairs have been found to have expression patterns associated with the same phenotype in both species: *mir-499*/*MYH7B* with heart development [79] and *mir-33*/*SREBF2* with cholesterol homeostasis [74], [75], [77]. Several independent studies in chicken have similarly indicated that *gga-mir-33* and its host gene *SREBF2* are highly expressed in the liver, suggesting involvement in expression upregulation of genes related to cholesterol biosynthesis [80], [81].

To further test the hypothesis that miRNA/host gene pairs with cross-species conserved co-location are coordinately expressed, we integrated expression data for 27 miRNA and their host genes using the GEA database. By comparing the gene expression data, we found that 24 miRNAs and their host genes had matching expression patterns in at least one disease (either over- or under-expression) (**Table S2**). Because of the same expression patterns and similar functions, the miRNA/host gene pairs are likely to be controlled by the same regulatory mechanisms. The miRNA/host gene pairs with conserved cross-species co-location, co-expression, and potential co-regulation provide a starting point for researchers investigating the involvement of intragenic miRNAs with disease development or control of production traits.

To better determine the role of the miRNA host genes from the pairs with conserved cross-species co-location, we performed a pathway enrichment analysis, using the IPA software [33]. Pathway analysis performed on the 23 host genes (**Table 1**) revealed networks associated with cancer, dermatological diseases and conditions, and hematological diseases (**Figure S6A**). Most significant biological functions included cancer, in addition to reproductive system diseases and infectious diseases. A molecular network diagram was constructed involving 14 miRNA host genes (*CTDSPL*, *C9orf3*, *COL27A1*, *EGFL7*, *HNRNPK*, *NFYC*, *PANK1*, *SLIT2*, *SLIT3*, *SMC4*, *SREBF2*, *TLN2*, *TRPM1*, and *WWP2*) which were found related to cancer, dermatologic and hematological diseases (**Figure S6B**). Within this network, several hubs were found encoding transcription factors, the largest two of which were *MYC* (v-myc myelocytomatosis viral oncogene homolog (avian)) and *TP53* (tumor protein p53), previously also linked with regulation of miRNA gene expression [82], [83].

#### 3.2. Epigenetically silenced miRNA genes located within host genes

Silenced expression of co-located miRNA and host genes might also be a subject of epigenetic regulation [27]. Namely, the proximal CpG islands located within their promoter or 5′UTR regions could epigenetically silence gene expression through DNA hypermethylation. In a recent study, 81.2% of protein-coding genes harboring miRNA genes in their 5′-end have been found located 500 bp downstream of CpG islands [84]. By performing a cross-section of 133 miRNA genes that have previously been found to be epigenetically regulated in cancer [85], we found that 30 are located within protein-coding, and 13 within ncRNA host genes, *i*.*e*. genes encoding for lincRNAs (**Figure 1**, **Table 2**). However, in order to determine the exact proportion of epigenetically regulated miRNA/host gene pairs a systematic genome-wide epigenetic analysis should be performed. Previous studies revealed that five miRNA genes as well as their host genes (*hsa-mir-10a*/*HOXB4*, *hsa-mir-126*/*EGFL7*, *hsa-mir-152*/*COPZ2*, *hsa-mir-191*/*DALRD3*, and *hsa-mir-342*/*EVL*) were found to be epigenetically downregulated, either by histone modification and/or CpG island hypermethylation in the promoter region in cancer cells [27], [86]–[89] (**Table 2**). Additionally, several host genes have, independently of miRNA studies, been found to be silenced through DNA hypermethylation: *DALRD3* [88], *HOXA9* [90]–[92], *HOXB4* [93], *HOXB7* [94], *HOXC4* [95], *HOXD3* [96], *HTR2C* [97], and *IGF2* [98]. The identified epigenetically regulated intragenic miRNA genes can now be analyzed together with their host genes in order to study their potential epigenetic co-regulation. We found that around half (20/43) of the epigenetically silenced miRNA genes were located within the 5′-UTR or in the first intron or exon of their host genes, suggesting the possibility of shared promoter regions that comprise CpG islands. Further studies on epigenetic regulation of miRNA/genes may reveal novel approaches for prevention or treatment of human cancer.

**Table 2:** Host genes for epigenetically silenced miRNA genes in cancer.

miRNA gene	location within host gene	host gene	study describing epigenetic regulation of host gene
***Protein-coding host genes***			
*hsa-let-7a-3*	exon 5	*RP4-695O20__B.10*	/
*hsa-mir-107*	intron 4–6	*PANK1*	/
*hsa-mir-10a*	intron 1, 5′-UTR	*HOXB3*	/
	5′-UTR	*HOXB4*	Zhend et al., 2009 [93], Shen et al., 2012 [89] *
*hsa-mir-10b*	intron 1	*HOXD3*	Kron et al., 2010 [96]
*hsa-mir-1-1*	intron 1, 2	*C20orf166*	/
*hsa-mir-126*	intron 5–7	*EGFL7*	Saito et al., 2009 [27] *
*hsa-mir-139*	intron 1–3	*PDE2A*	/
*hsa-mir-140*	intron 3, 6, 7, 9, 13, 14, 16	*WWP2*	/
*hsa-mir-148b*	intron 1, 2	*COPZ1*	/
*hsa-mir-152*	intron 1, 2	*COPZ2*	Tsuruta et al., 2011 [87] *
*hsa-mir-188*	intron 3	*CLCN5*	/
*hsa-mir-191*	intron 1	*DALRD3*	He et al., 2011 [88] *
*hsa-mir-196a-1*	intron 1	*HOXB7*	Bennett et al., 2009 [94]
*hsa-mir-196b*	exon 1–3	*HOXA9*	Bandyopadhyay et al., 2012 [92], Hwang et al., 2011 [90], Wu et al., 2007 [91]
	intron 1	*RP1-170O19.20*	/
*hsa-mir-198*	3′-UTR	*FSTL1*	/
*hsa-mir-204*	intron 3–7	*TRPM3*	/
*hsa-mir-23b*	intron 4–6, 14, 15	*C9orf3*	/
*hsa-mir-24-1*	intron 4, 5, 14, 15, exon 7	*C9orf3*	/
*hsa-mir-25*	intron 2, 4, 8, 12, 13	*MCM7*	/
*hsa-mir-27b*	intron 4–6, 14, 15	*C9orf3*	/
*hsa-mir-342*	intron 2–4	*EVL*	Grady et al., 2008 [86] *
*hsa-mir-425*	intron 1	*DALRD3*	He et al., 2011 [88]
*hsa-mir-448*	intron 4, 5	*HTR2C*	Anderton et al., 2008 [97]
*hsa-mir-483*	intron 2, 3, 5	*IGF2*	Dejeux et al., 2009 [98]
	intron 5	*INS-IGF2*	/
*hsa-mir-548c-1*	intron 14–16	*ATAD2*	/
*hsa-mir-570*	intron 3	*MUC20*	/
*hsa-mir-582*	intron 1–3	*PDE4D*	/
*hsa-mir-615*	intron 1	*HOXC5*	/
	intron 1	*HOXC4*	Issa, 2009 [95]
*hsa-mir-744*	intron 1–5	*MAP2K4*	/
*hsa-mir-9-1*	intron 1, 2	*C1orf61*	/
***Non-coding RNA genes*** * (gene type according to Ensembl)*			
*hsa-mir-124-1*	exon 1, 3, 4	*LINC00599* (lincRNA)	/
*hsa-mir-124-2*	intron 1	*RP11-32K4.2* (lincRNA)	/
*hsa-mir-137*	exon 3	*MIR137HG* (lincRNA)	/
*hsa-mir-17*	exon 3, intron 3	*MIR17HG* (lincRNA)	/
*hsa-mir-193b*	intron 1	*RP11-65J21.3* (lincRNA)	/
*hsa-mir-205*	exon 2, 4, intron 2, 3	*MIR205HG* (lincRNA)	/
*hsa-mir-20a*	exon 3, intron 3	*MIR17HG* (lincRNA)	/
*hsa-mir-30a*	intron 3	*LINC00472* (lincRNA)	/
*hsa-mir-31*	intron 1	*MIR31HG* (lincRNA)	/
*hsa-mir-370*	intron 5	*MEG8* (lincRNA)	/
*hsa-mir-9-2*	exon 3, 4, intron 2, 3	*LINC00461* (lincRNA)	/
*hsa-mir-9-3*	intron 1	*CTD-2335A18.1* (lincRNA)	/
*hsa-mir-99a*	intron 1, 3, 5, 6	*LINC00478* (lincRNA)	/

/− host gene not found to be regulated by DNA methylation in references.

* - studies describing epigenetically regulated host gene and resident miRNA gene.

Host gene names: *ATAD2*: ATPase family, AAA domain containing 2; *C1orf61*: chromosome 1 open reading frame 61; *C20orf166*: chromosome 20 open reading frame 166; *CLCN5*: chloride channel, voltage-sensitive 5; *COPZ1*: coatomer protein complex, subunit zeta 1; *COPZ2*: coatomer protein complex, subunit zeta 2; *DALRD3*: DALR anticodon binding domain containing 3; *EVL*: Enah/Vasp-like; *FSTL1*: follistatin-like 1; *HOXA9*: homeobox A9; *HOXB4*: homeobox B4; *HOXB7*: homeobox B7; *HOXC4*: homeobox C4; *HOXC5*: homeobox C5; *HOXD3*: homeobox D3; *HTR2C*: 5-hydroxytryptamine (serotonin) receptor 2C, G protein-coupled; *IGF2*: insulin-like growth factor 2 (somatomedin A); *INS-IGF2*: INS-IGF2 readthrough; *LINC00461*: long intergenic non-protein coding RNA 461; *LINC00478*: long intergenic non-protein coding RNA 478; *LINC00472*: long intergenic non-protein coding RNA 472; *MAP2K4*: mitogen-activated protein kinase kinase 4; *MCM7*: minichromosome maintenance complex component 7; *MIR137HG:* mir-137 host gene (non-protein coding); *MEG8*: maternally expressed 8 (non-protein coding); *MIR17HG*: mir-17–92 cluster host gene (non-protein coding); *MIR205HG*: mir-205 host gene (non-protein coding); *MIR31HG*: mir-31 host gene (non-protein coding); *MUC20*: mucin 20, cell surface associated; *PDE2A*: phosphodiesterase 2A, cGMP-stimulated; *PDE4D*: phosphodiesterase 4D, cAMP-specific; *TRPM3*: transient receptor potential cation channel, subfamily M, member 3.

### 4. MicroRNA/host Gene Pairs – Potential for Misattribution of Phenotype?

In our study we demonstrated that a very large proportion of miRNAs are located within the host genes (**Figure 2**) in human (1,131/1,600), mouse (518/855), and chicken (240/499) and that miRNA/host gene pairs have important conservation and co-expression issues. Our study can be used as a platform for researchers to re-examine questions related to earlier or planned studies correlating genetic variation or modification of the miRNA/host gene pairs with diseases or trait control. Namely, it is prudent to ask if some of the gene variation-phenotype association studies targeted at the miRNA host genes, spontaneous, radiation or chemically induced mutations, knockout and overexpression models need reinterpretation to take into account collateral effects on miRNAs. MicroRNA genes harbored within another host gene, as shown by many examples in our study, may have several target genes and functions unrelated to their host genes. The host gene mutations or modifications may also collaterally affect the level, time or tissue specificity of miRNA expression thereby leading to several pleiotropic effects in the phenotype that could not be causally ascribed to the host gene only. Many types of spontaneous and induced mutations within the host gene locus (*e.g*. promoter, splicing mutations, or mRNA stability mutations) may affect the transcript quantity, temporal and/or spatial expression pattern of hosted miRNA.

In addition to aforementioned effects, transgenic overexpression and knockout host gene models may alter hosted miRNA function through exogenous sequences left in the locus such as selection marker genes (*e.g.* neomycin resistance, *NeoR*), plasmid vector and other sequences (*e.g.* strong phosphoglycerate kinase (*pgk*) gene promotor). We note that among the knockout mice of relevance in **Tables 1A and B**, most models retained the *NeoR* marker and also other exogenous sequences that can potentially affect expression and function of hosted miRNA gene in addition to the target host gene itself. Many targeting constructs are designed to delete large portions of the target gene in order to ensure loss of function of the host locus. The weakness of this strategy is that some of the deleted sequence may contain miRNAs or regulatory sequences affecting neighboring genes. Significantly for this discussion, inadvertent deletion of *mmu-mir-126* has led to the misattribution of phenotype - angiogenesis defects previously reported in a knockout of the *Egfl7* locus were subsequently shown to have arisen due to deletion of the *mmu-mir-126* [**99**].

A degree of common sense can be applied to assessing the level of confidence attributed to specific phenotypes of the miRNA/host gene pairs. Where the phenotype is consistent with what was expected from knowledge of gene expression and biochemistry for the host gene and hosted miRNA gene, one can be reasonably comfortable in attributing a phenotype to the host target gene function. However, where the phenotype is unexpected, or where multiple genotype-phenotype or multiple gene modification models show disparate effects, then one is justified in being more cautious and to proceed by further experimentation to differentiate the host gene from hosted miRNA gene phenotypic effects. In the future gene modification experiments many concerns raised above can be minimized by using recent technology of Zinc finger [100] and Tal nucleases [101]. These methods generate minimal targeted modifications (*i.e.* point mutation generating premature stop codon) and do not leave exogenous sequence in the genome thereby providing excellent transgenic *in vitro* and *in vivo* models for miRNA/host gene pairs studies.

Our web site (http://www.integratomics-time.com/miR-host/) provides an efficient tool to check which host genes contain miRNAs while other tables list important functional and literature information to aid researchers in re-examining potential misattribution of phenotype previously ascribed to host genes or hosted miRNA genes only.

### 5. Future Perspective

Our assembled and supplemented catalog of miRNA/host gene pairs available via the web application will provide researchers with a data mining tool for investigating miRNA/host gene pair involvement of their coordinated expression, shared regulation, and function in diseases: 1) structural annotation - miRNA/host gene pairs with conserved cross-species co-location in the examined species present candidate genes for future annotation in other species. 2) Functional annotation - miRNA/host gene pairs with matching expression patterns integrated from databases are high priority candidates for experimental validation of their potential co-expression and co-regulation. 3) MicroRNAs overlapping with protein-coding and other ncRNA host genes (lincRNA and snoRNA) present candidates for evaluating molecular mechanisms underlying previously shown functional links. 4) MicroRNAs residing within genes encoding for miRNA silencing machinery present important miRNA-related regulatory cross-talk needing additional mechanistic experimentation to elucidate targeting interplay in which miRNAs target genes for miRNA processing components and, in a feedback loop, influences the production of miRNAs. 5) Identification and validation of polymorphisms located within miRNA genes, their host genes, and genes encoding for and processing machinery components may also reveal whether they contribute to phenotypic variation, including disease susceptibility. 6) Epigenetic silencing of both, miRNA and their host genes, offers insights into their shared regulation and their re-expression may be used to contribute to the effects of epigenetic therapy. The assembled epigenetically regulated intragenic miRNAs represent candidate genes for the study of miRNA/host gene pair epigenetic co-regulation. 7) Our web site also provides an efficient tool to identify certain miRNA/host gene pairs where previous studies show inconsistencies of the effects of natural or induced mutations on the phenotype. We point to examples where such phenotype misinterpretations could arise due to attribution collateral effects of such mutations on hosted miRNAs. Our catalog can therefore direct researchers to critically examine designs and interpretation of such miRNA/host gene cases.

### Conclusion

In conclusion, the assembled catalog is, to our knowledge, the most comprehensive integrated assembly of intragenic miRNAs and their host genes in human, mouse, and chicken. The systematically integrated physical (genomic location and cross-species conserved co-location) and functional characterization (co-expression data) of miRNA/host gene pairs provides a starting point for researchers investigating involvement of intragenic miRNAs with human and animal health, and animal production traits. Using this approach we found that miRNA/host gene pairs with cross-species conserved co-location are very likely to be co-expressed. The expanding field of miRNA research requires a consideration of interplay of interconnecting regulatory mechanisms and their function into an intricate network, in which miRNA genes and their co-expressed host genes also play a role.

## Supporting Information

Figure S1 **Print-screen of** **genomic view of intragenic miRNAs in human.** Enlarged chromosome 22 showing *hsa-mir-1306* and its host gene *DGCR8* with databases linked through outgoing links.
(TIF)

Figure S2 **Distribution of intragenic miRNA genes according to chromosome in A) human, B) mouse, and C) chicken.**
(PDF)

Figure S3 **MicroRNA genes located within genes encoding for the miRNA processing machinery in mouse.**
(TIF)

Figure S4 **Venn diagram of the number of miRNA/host gene pairs with cross-species conserved co-location.**
(TIF)

Figure S5 **Alignment of orthologous miRNA genes.** **A**) Human (*hsa-mir-3064*) and mouse (*mmu-mir-3064*) miRNA genes matching the sequence in chicken. Mature miRNA regions are marked with a square. **B**) Murine miRNA genes (*mmu-mir-677*, *-686*, -*717*, -*763*, -*1839*, -*1893*, -*1896*, -*1897*, -*1898*, -*1902*, -*1907*, -*1949*, -*2139*, -*3059*, and -*5125*) aligned with human sequences. **C**) Fifteen potential human miRNA genes acquired based on alignment with 15 murine miRNA genes. **D**) Small RNA expression data for sequences matching the four potential new miRNA genes in human (*hsa-mir-677*, -*1839*, -*1897*, and -*1949*).
(DOC)

Figure S6 **Network analysis of host genes from 27 conserved miRNA/host gene pairs, in human and mouse.** **A)** Top network and biological functions associated miRNA host genes. **B)** Diagram of a top molecular network showing 14 miRNA host genes (gray-filled shapes) associated with cancer, dermatological diseases and conditions, and hematological diseases. White-filled shapes indicate connecting elements in between host genes in the network.
(DOCX)

Table S1 **Intragenic miRNAs with polymorphic seed regions in human, mouse, and chicken.**
(DOC)

Table S2 **Dysregulation of expression in diseases associated with 27 human miRNA/host gene pairs with cross-species conserved co-location.**
(DOCX)
