# Supplementary figures and images for: Genome-Wide and Species-Wide In Silico Screening for Intragenic MicroRNAs in Human, Mouse and Chicken
Source: PLoS One. 2013 Jun 6;8(6):e65165. doi: 10.1371/journal.pone.0065165 (PMC3675212; doi:10.1371/journal.pone.0065165)

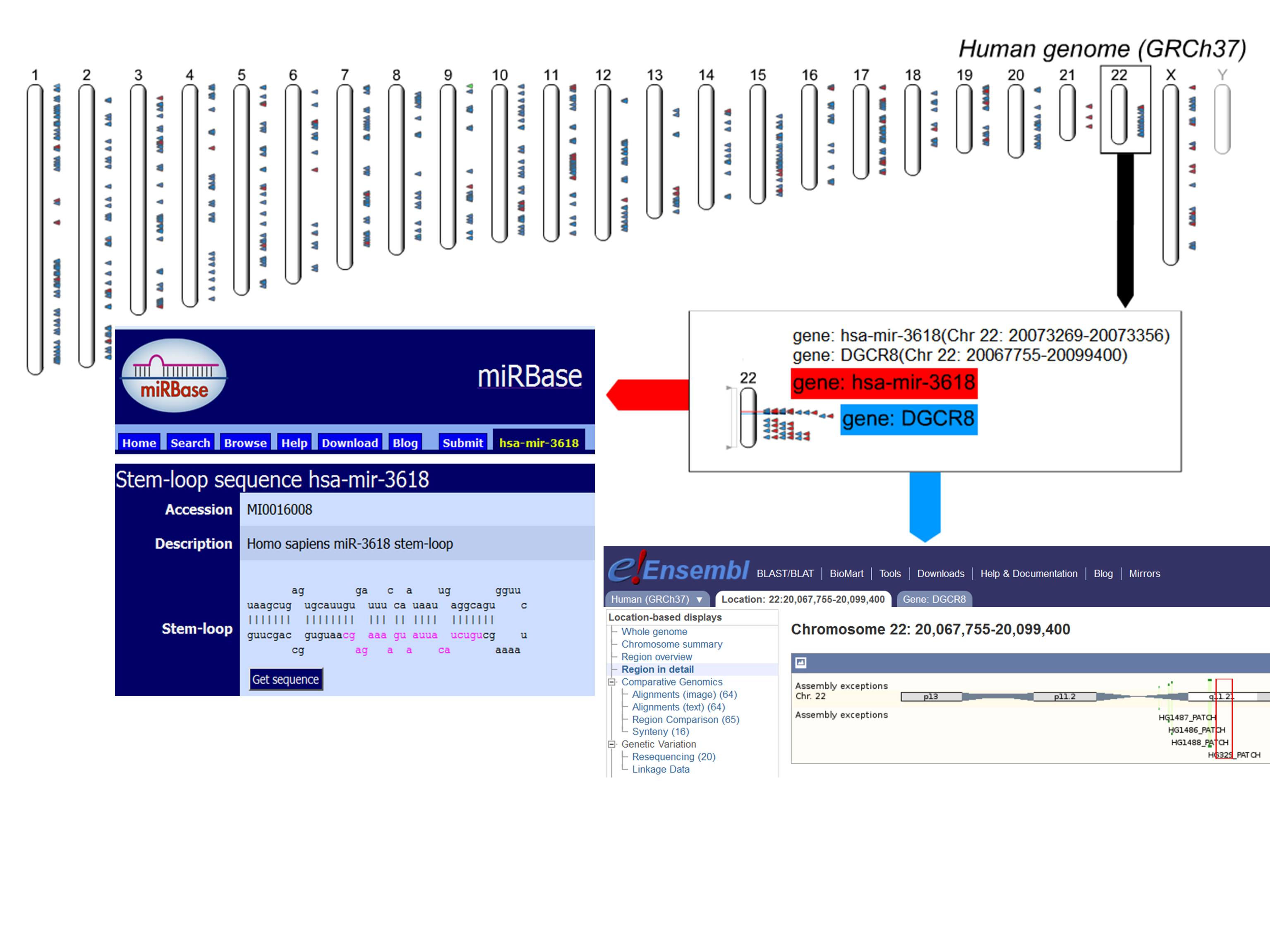

Supplement: Figure S1 — Print-screen of genomic view of intragenic miRNAs in human. Enlarged chromosome 22 showing hsa-mir-1306 and its host gene DGCR8 with databases linked through outgoing links. (TIF) [file pone.0065165.s001.tif]

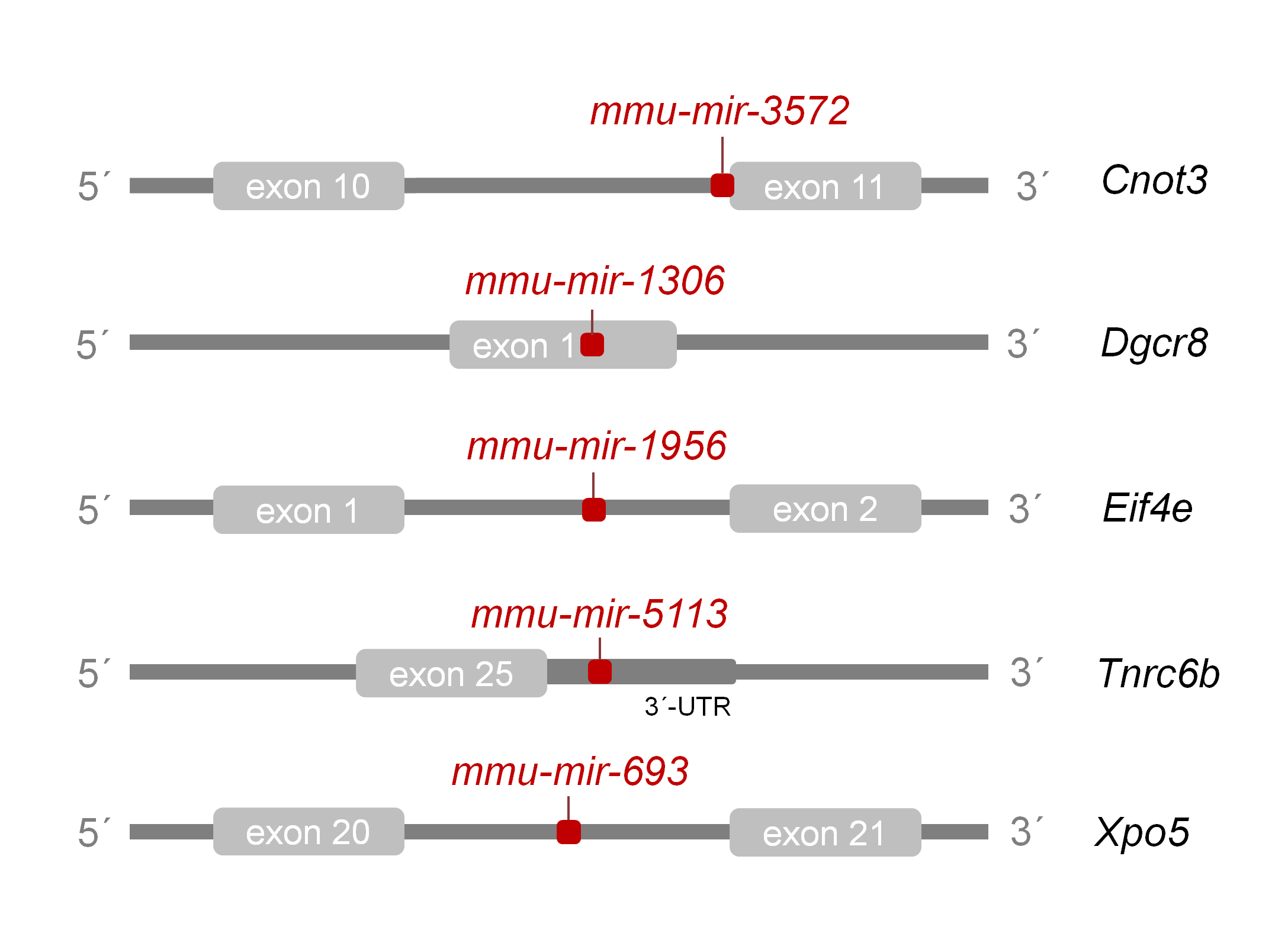

Supplement: Figure S3 — MicroRNA genes located within genes encoding for the miRNA processing machinery in mouse. (TIF) [file pone.0065165.s003.tif]

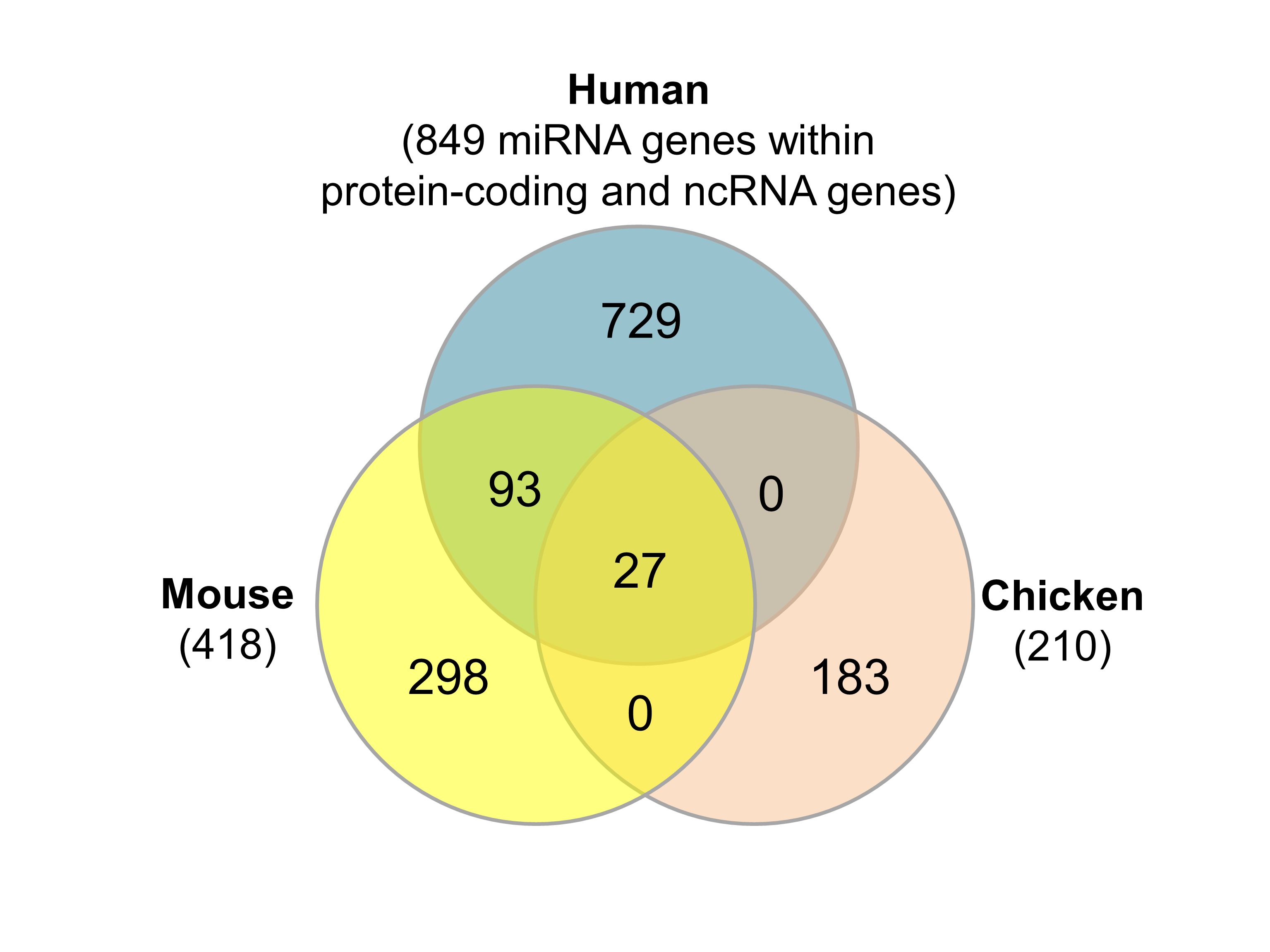

Supplement: Figure S4 — Venn diagram of the number of miRNA/host gene pairs with cross-species conserved co-location. (TIF) [file pone.0065165.s004.tif]

**

**

**A**

**
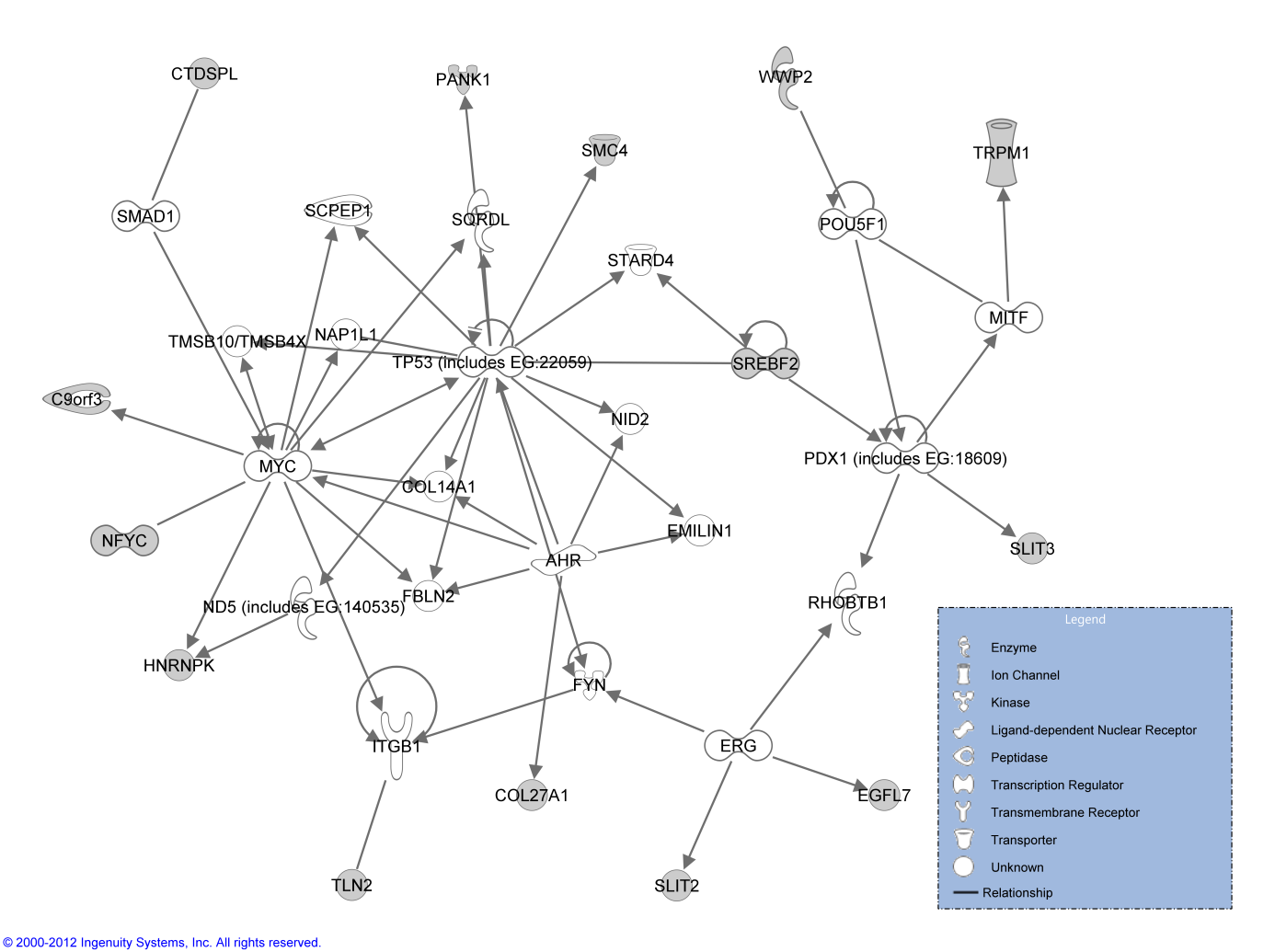
**

**B**

Supplement: Figure S6 — Network analysis of host genes from 27 conserved miRNA/host gene pairs, in human and mouse. A) Top network and biological functions associated miRNA host genes. B) Diagram of a top molecular network showing 14 miRNA host genes (gray-filled shapes) associated with cancer, dermatological diseases and conditions, and hematological diseases. White-filled shapes indicate connecting elements in between host genes in the network. (DOCX) [file pone.0065165.s006.docx]
