# Supplementary material for: Genome-Wide and Species-Wide In Silico Screening for Intragenic MicroRNAs in Human, Mouse and Chicken
Source: PLoS One. 2013 Jun 6;8(6):e65165. doi: 10.1371/journal.pone.0065165 (PMC3675212; doi:10.1371/journal.pone.0065165)
Supplement: Figure S2 — Distribution of intragenic miRNA genes according to chromosome in A) human, B) mouse, and C) chicken. (PDF) [file pone.0065165.s002.pdf]

Supporting Figure S2: Distribution of intragenic miRNA genes according to chromosome in **A**) human, **B**) mouse, and **C**) chicken.

A

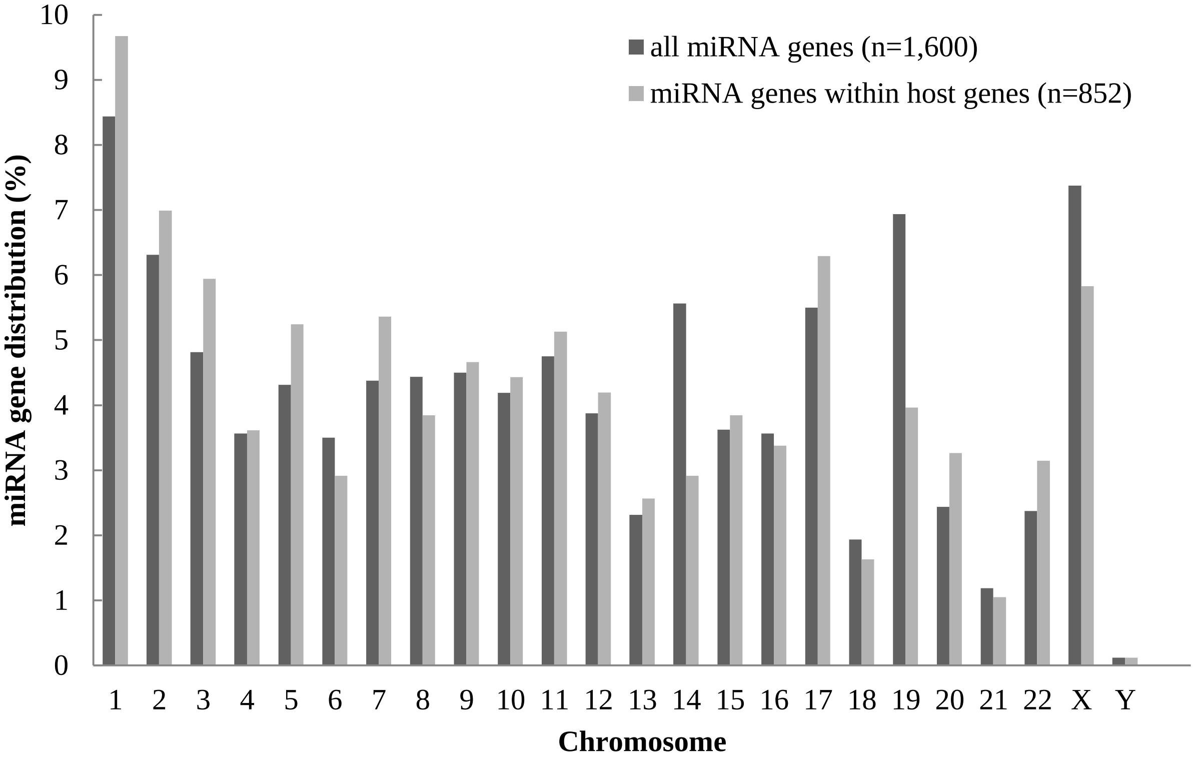

B

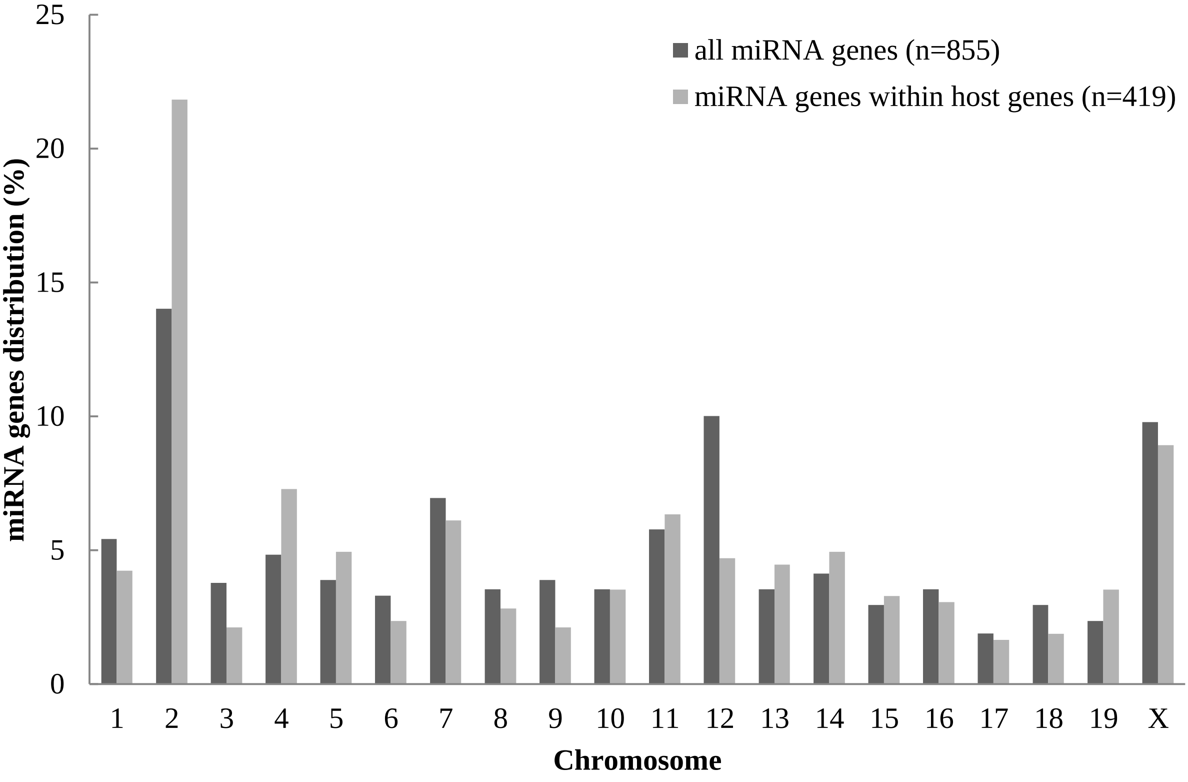

C

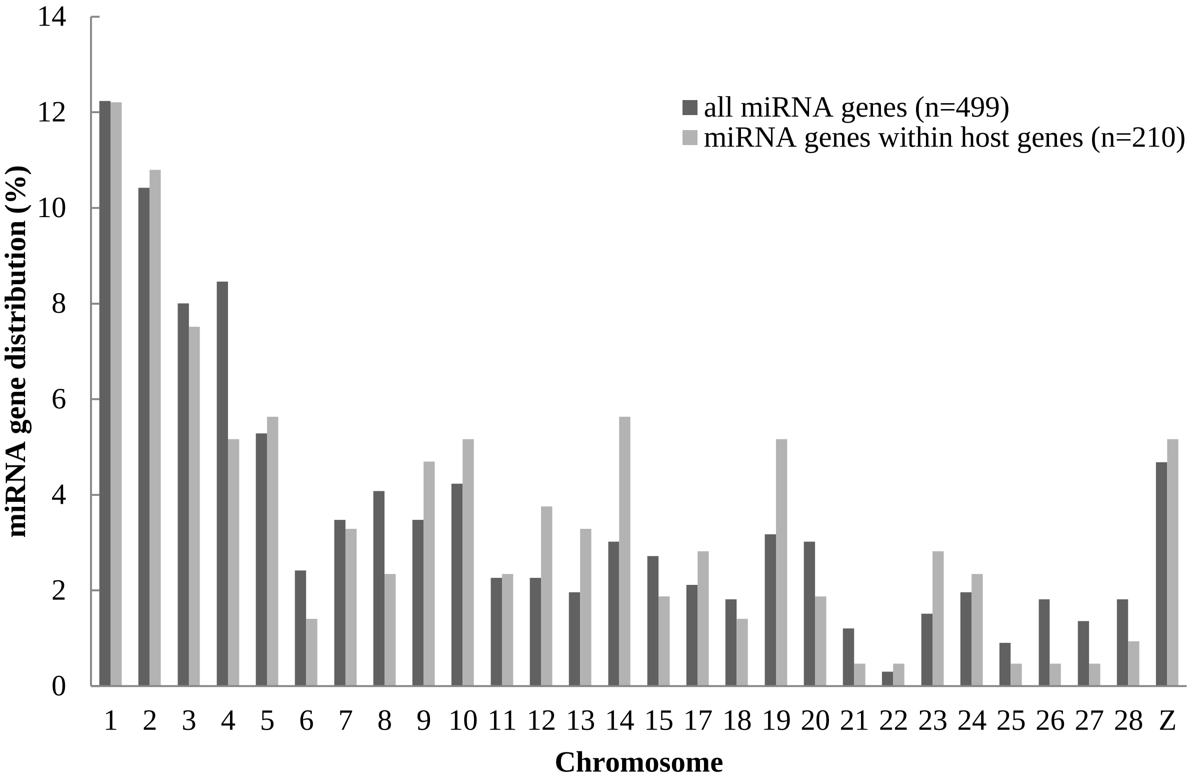

14
12
10
8
6
4
2
0
miRNA gene distribution (%)
all miRNA genes (n=499)
miRNA genes within host genes (n=210)
1 2 3 4 5 6 7 8 9 10 11 12 13 14 15 17 18 19 20 21 22 23 24 25 26 27 28 Z
Chromosome
