# Supplementary material for: Genome-Wide and Species-Wide In Silico Screening for Intragenic MicroRNAs in Human, Mouse and Chicken
Source: PLoS One. 2013 Jun 6;8(6):e65165. doi: 10.1371/journal.pone.0065165 (PMC3675212; doi:10.1371/journal.pone.0065165)
Supplement: Figure S5 — Alignment of orthologous miRNA genes. A) Human (hsa-mir-3064) and mouse (mmu-mir-3064) miRNA genes matching the sequence in chicken. Mature miRNA regions are marked with a square. B) Murine miRNA genes (mmu-mir-677, -686, -717, -763, -1839, -1893, -1896, -1897, -1898, -1902, -1907, -1949, -2139, -3059, and -5125) aligned with human sequences. C) Fifteen potential human miRNA genes acquired based on alignment with 15 murine miRNA genes. D) Small RNA expression data for sequences matching the four potential new miRNA genes in human (hsa-mir-677, -1839, -1897, and -1949). (DOC) [file pone.0065165.s005.doc]

**Supporting Figure S5:** Alignment of orthologous miRNA genes. **A**) Human (*hsa-mir-3064*) and mouse (*mmu-mir-3064*) miRNA genes matching the sequence in chicken. Mature miRNA regions are marked with a square. **B**) Murine miRNA genes (*mmu-mir-677*, *-686*, -*717*, -*763*, -*1839*, -*1893*, -*1896*, -*1897*, -*1898*, -*1902*, -*1907*, -*1949*, -*2139*, -*3059*, and -*5125*) aligned with human sequences. **C**) Fifteen potential human miRNA genes acquired based on alignment with 15 murine miRNA genes. **D**) Small RNA expression data for sequences matching the four potential new miRNA genes in human (*hsa-mir-677*, -*1839*, -*1897*, and -*1949*).


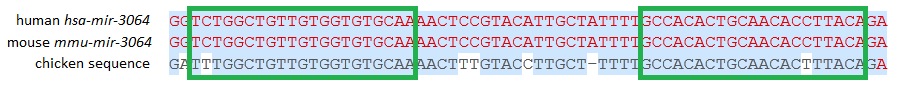


**A**

**
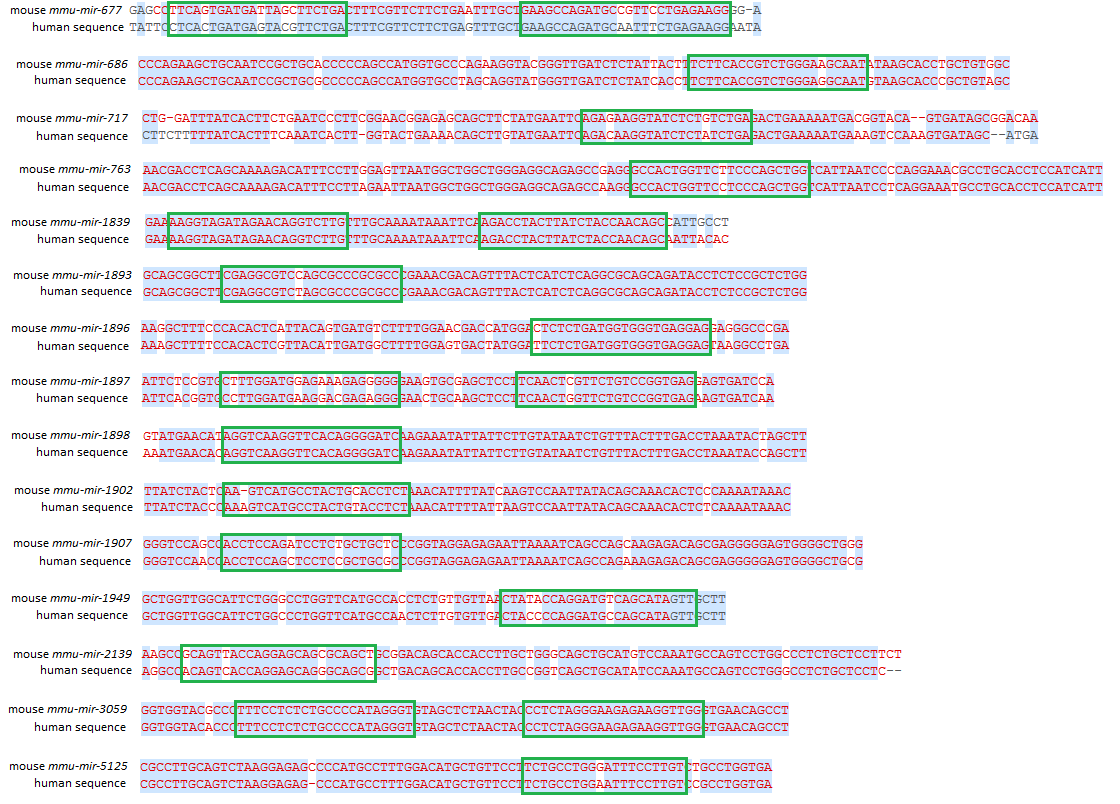
**

**B**

| MOUSE | | HUMAN (potential miRNA) | | | | |
| --- | --- | --- | --- | --- | --- | --- |
| miRNA gene | host gene | proposed miRNA gene name | chromosome (orientation) | location- start | location- end | host gene |
| *mmu-mir-2139* | *Klhdc7* | *hsa-mir-2139* | 1 (-) | 18807486 | 18807578 | *KLHDC7A* |
| *mmu-mir-717* | *Gpc3* | *hsa-mir-717* | X (+) | 132880769 | 132880877 | *GPC3* |
| *mmu-mir-1902* | *Hipk3* | *hsa-mir-1902* | 11 (-) | 33376011 | 33376091 | *HIPK3* |
| *mmu-mir-1898* | *Zfr* | *hsa-mir-1898* | 5 (-) | 32379501 | 32379583 | *ZFR* |
| *mmu-mir-1893* | *Epc1* | *hsa-mir-1893* | 10 (+) | 32635792 | 32635874 | *EPC1* |
| *mmu-mir-686* | *Psmb5* | *hsa-mir-686* | 14 (+) | 23502739 | 23502847 | *PSMB5* |
| *mmu-mir-763* | *Hmga2* | *hsa-mir-763* | 12 (-) | 66251793 | 66251912 | *HMGA2* |
| *mmu-mir-1907* | *Trps1* | *hsa-mir-1907* | 8 (+) | 116680187 | 116680276 | *TRPS1* |
| *mmu-mir-1839* | *AC167122.1* (snoRNA) | *hsa-mir-1839* | 15 (+) | 83424697 | 83424823 | *SCARNA15* (snoRNA) |
| *mmu-mir-3059* | *Mgat4c* | *hsa-mir-3059* | 12 (-) | 87138845 | 87138925 | *RP11-202H2.1* (lincRNA upstream of *MGAT4C*) |
| *mmu-mir-5125* | *Srrm2* | *hsa-mir-5125* | 16 (+) | 2819873 | 2819950 | *SRRM2* |
| *mmu-mir-677* | *Atp5b* | *hsa-mir-677* | 12 (-) | 57037464 | 57037535 | *ATP5B* |
| *mmu-mir-1896* | *4930470G03Rik* (lincRNA) | *hsa-mir-1896* | 6 (-) | 28244564 | 28244644 | *RP5-874C20.3* (pseudogene) |
| *mmu-mir-1897* | *Sox2ot* (non-coding) | *hsa-mir-1897* | 3 (+) | 181417493 | 181417571 | *SOX2-OT* (lincRNA) |
| *mmu-mir-1949* | *SNORA74.2* (snoRNA) | *hsa-mir-1949* | 5 (+) | 138611869 | 138612009 | *SNORA74.5* (snoRNA)*, MATR3* |

**C**

**
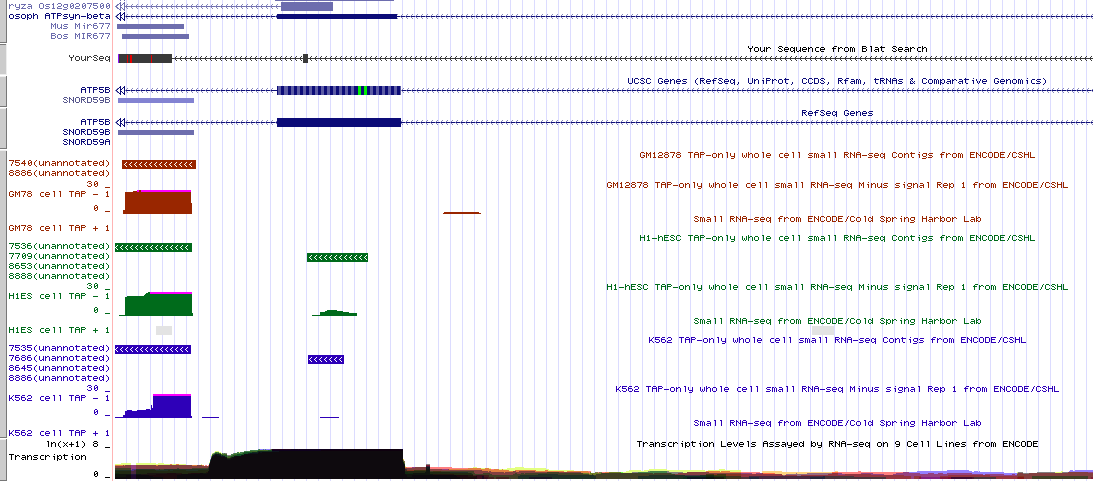
**

*hsa-mir-677*


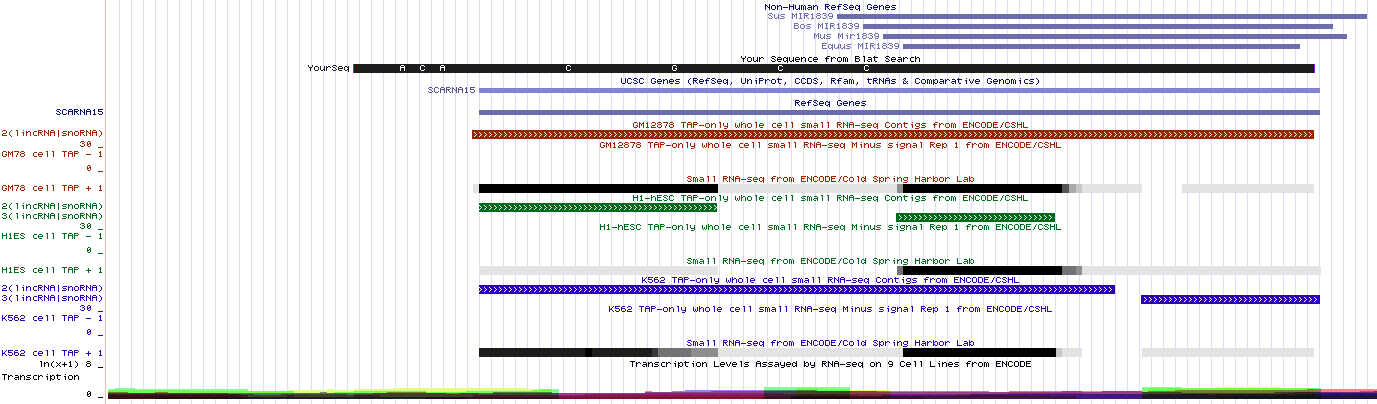


*hsa-mir-1839*


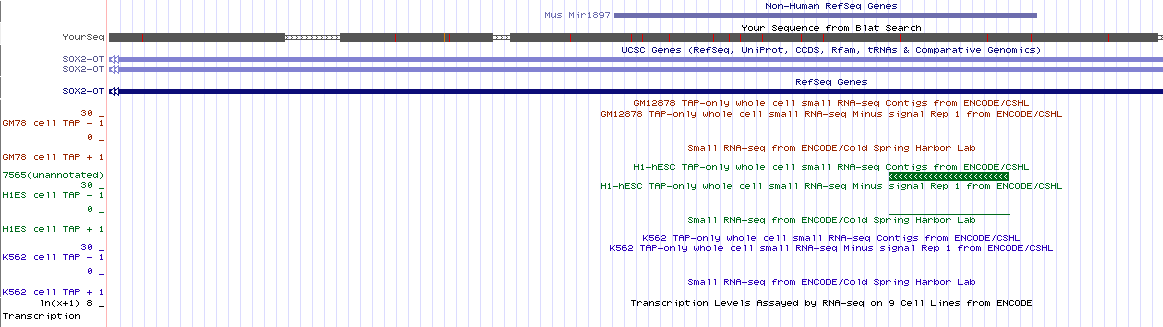


*hsa-mir-1897*


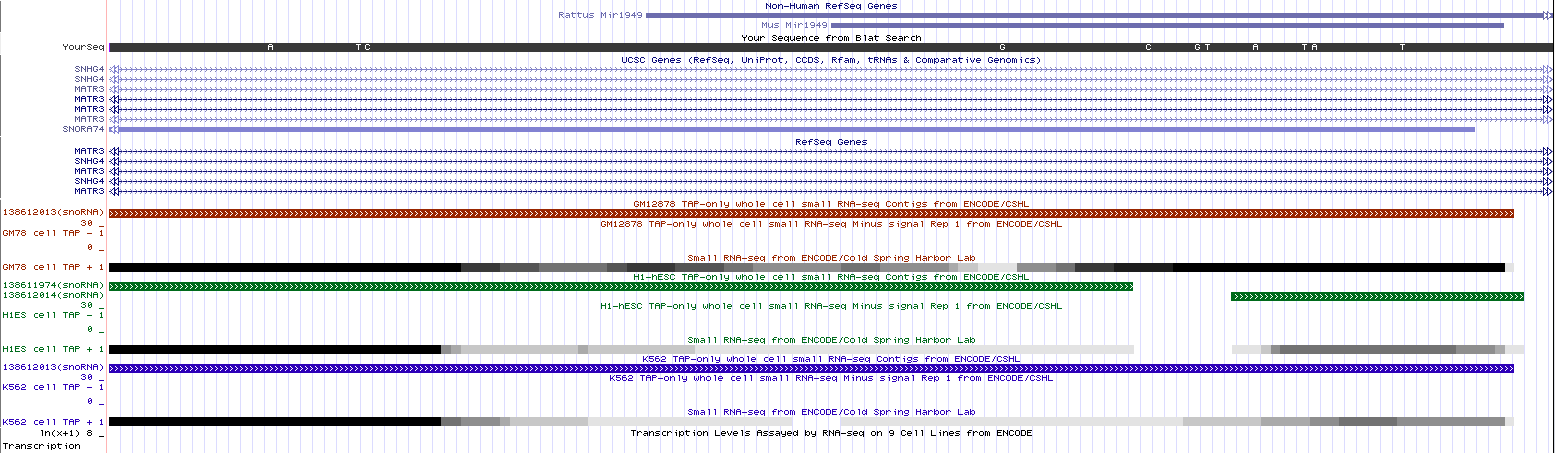


*hsa-mir-1949*

**D**
