# Supplementary material for: Genome-Wide and Species-Wide In Silico Screening for Intragenic MicroRNAs in Human, Mouse and Chicken
Source: PLoS One. 2013 Jun 6;8(6):e65165. doi: 10.1371/journal.pone.0065165 (PMC3675212; doi:10.1371/journal.pone.0065165)
Supplement: Table S1 — Intragenic miRNAs with polymorphic seed regions in human, mouse, and chicken. (DOC) [file pone.0065165.s007.doc]

**Supporting Table S1**: **Intragenic miRNAs with polymorphic seed regions in human, mouse, and chicken.**

| **miRNA gene** | **host gene ID** | **location of miRNA gene within host gene** | **SNP ID** | **nucleotide substitution** |
| --- | --- | --- | --- | --- |
| ***Validated polymorphisms – HUMAN*** | | | | |
| ***hsa-mir-15b*** | *SMC4* | intron | [rs192595529](http://www.ncbi.nlm.nih.gov/projects/SNP/snp_ref.cgi?rs=rs192595529) | G>A |
| ***hsa-mir-33a*** | *SREBF2* | intron | rs77809319 | A>G |
| ***hsa-mir-146a*** | *CTC-231O11.1* | exon | rs2910164 | C>G |
| ***hsa-mir-188*** | *CLCN5* | intron | [rs191840972](http://www.ncbi.nlm.nih.gov/projects/SNP/snp_ref.cgi?rs=rs191840972) | C>T |
| ***hsa-mir-449c*** | *CDC20B* | intron | rs35770269 | A>T |
| ***hsa-mir-488*** | *ASTN1* | intron | [rs186200318](http://www.ncbi.nlm.nih.gov/projects/SNP/snp_ref.cgi?rs=rs186200318) | T>C |
| ***hsa-mir-499a*** | *MYH7B* | intron | rs3746444 | A>G |
|  |  |  | [rs150018420](http://www.ncbi.nlm.nih.gov/projects/SNP/snp_ref.cgi?rs=rs150018420) | C>T |
| ***hsa-mir-501*** | *CLCN5* | intron | [rs149912461](http://www.ncbi.nlm.nih.gov/projects/SNP/snp_ref.cgi?rs=rs149912461) | A>G |
| ***hsa-mir-505*** | *ATP11C* | intron | [rs143213653](http://www.ncbi.nlm.nih.gov/projects/SNP/snp_ref.cgi?rs=rs143213653) | C>T |
| ***hsa-mir-548aa-2*** | *PITPNC1* | intron | [rs149172942](http://www.ncbi.nlm.nih.gov/projects/SNP/snp_ref.cgi?rs=rs149172942) | C>T |
| ***hsa-mir-548al*** | *RP11-702H23.4* | intron | rs515924 | A>G |
| ***hsa-mir-548ao*** | *SFRP1* | intron | rs79091838 | G>A |
|  |  |  | [rs150751643](http://www.ncbi.nlm.nih.gov/projects/SNP/snp_ref.cgi?rs=rs150751643) | C>A |
| ***hsa-mir-548t*** | *GALNT7* | intron | rs73872515 | A>C |
| ***hsa-mir-551a*** | *MEGF6* | intron | [rs187195064](http://www.ncbi.nlm.nih.gov/projects/SNP/snp_ref.cgi?rs=rs187195064) | C>T |
| ***hsa-mir-553*** | *RTCA* | intron | [rs190622705](http://www.ncbi.nlm.nih.gov/projects/SNP/snp_ref.cgi?rs=rs190622705) | A>G |
| ***hsa-mir-562*** | *DIS3L2* | intron | [rs140596642](http://www.ncbi.nlm.nih.gov/projects/SNP/snp_ref.cgi?rs=rs140596642) | CTGTACCATTTGCACTCC > - |
| ***hsa-mir-574*** | *FAM114A1* | intron | rs147472159 | GT>- |
| ***hsa-mir-575*** | *SCD5* | intron | rs149186367 | C>T |
| ***hsa-mir-585*** | *SLIT3* | intron | rs62376935 | C>T |
| ***hsa-mir-590*** | *EIF4H* | intron | rs189727189 | C>T |
| ***hsa-mir-593*** | *SND1* | intron | rs73721294 | C>T |
| ***hsa-mir-604*** | *SVIL* | intron | rs186844507 | C>T |
| ***hsa-mir-605*** | *PRKG1* | intron | [rs113212828](http://www.ncbi.nlm.nih.gov/sites/entrez?db=snp&cmd=search&term=rs113212828) | A>G |
| ***hsa-mir-627*** | *VPS39* | intron | rs2620381 | T>G |
| ***hsa-mir-642a*** | *GIPR* | intron | rs78902025 | T>G |
| ***hsa-mir-660*** | *CLCN5* | intron | rs180785970 | G>C |
| ***hsa-mir-664b*** | *DKC1* | intron | rs112159031 | G>A |
| ***hsa-mir-933*** | *ATF2* | intron | rs139770589 | T>C |
| ***hsa-mir-938*** | *SVIL* | intron | rs12416605 | G>A |
| ***hsa-mir-940*** | *RP11-304L19.13* | intron | rs149527765 | G>A |
| ***hsa-mir-941-3*** | *DNAJC5* | intron | rs113672516 | A>G |
|  |  |  | rs35544770 | A>G |
| ***hsa-mir-941-4*** | *DNAJC5* | intron | rs35544770 | A>G |
| ***hsa-mir-943*** | *WHSC2* | exon | rs186568343 | C>A |
| ***hsa-mir-1178*** | *CIT* | exon | rs7311975 | T>C |
| ***hsa-mir-1227*** | *PLEKHJ1* | intron | rs112440628 | C>G |
| ***hsa-mir-1233-1*** | *GOLGA8A* | intron | rs71309450 | ->G |
| ***hsa-mir-1234*** | *CPSF1* | intron | rs75169642 | C>G |
|  |  |  | rs2291134 | C>G |
| ***hsa-mir-1236*** | *RDBP* | intron | rs185147690 | G>C |
| ***hsa-mir-1237*** | *RPS6KA4* | intron | rs113909793 | G>A |
| ***hsa-mir-1254-1*** | *CCAR1* | intron | rs138812323 | G>A |
| ***hsa-mir-1255b-2*** | *DCAF6* | intron | rs189709980 | G>C |
| ***hsa-mir-1276*** | *KLHL25* | intron | rs34381260 | ->A |
| ***hsa-mir-1292*** | *NOP56* | intron | rs73576045 | C>T |
| ***hsa-mir-1304*** | *TAF1D* | exon | rs76857625 | A>G |
|  |  | intron | rs79759099 | A>G |
| ***hsa-mir-1322*** | *PINX1* | intron | rs189895541 | A>G |
|  | *SOX7* | intron | rs113859132 | G>A |
| ***hsa-mir-1469*** | *NR2F2* | intron | rs116596918 | G>C |
| ***hsa-mir-2392*** | *MEG3* | intron | rs118055959 | A>G |
| ***hsa-mir-2682*** | *MIR137HG* | intron | rs74904371 | C>T |
| ***hsa-mir-2909*** | *AATF* | intron | rs187707102 | G>A |
| ***hsa-mir-3116-1*** | *INADL* | intron | rs192207383 | G>A |
| ***hsa-mir-3117*** | *SGIP1* | intron | rs12402181 | G>A |
| ***hsa-mir-3118-2*** | *BX571672.1 BX571672.4* | intron | rs61786895 | G>A |
| ***hsa-mir-3126*** | *ANTXR1* | intron | rs182148652 | G>A |
|  |  |  | rs187256070 | A>T |
| ***hsa-mir-3157*** | *RP11-429G19.2* | intron | rs141770574 | G>A |
| ***hsa-mir-3161*** | *PTPRJ* | intron | rs113098367 | ->A |
|  |  |  | rs11382316 | ->A |
| ***hsa-mir-3196*** | *BIRC7* | intron | rs113297757 | G>A |
| ***hsa-mir-3611*** | *CUL2* | intron | rs150577768 | A>C |
| ***hsa-mir-3614*** | *TRIM25* | exon | rs118080115 | T>C |
| ***hsa-mir-3615*** | *SLC9A3R1* | exon | rs112977728 | C>T |
| ***hsa-mir-3620*** | *ARF1* | intron | rs2070960 | C>T |
| ***hsa-mir-3655*** | *IK* | 3’-UTR | rs146400503 | C>T |
| ***hsa-mir-3666*** | *FOXP2* | intron | rs186005043 | T>C |
| ***hsa-mir-3677*** | *RP11-304L19.13* | intron | rs138871259 | G>A |
| ***hsa-mir-3682*** | *ASB3* | intron | rs116380885 | T>C |
| ***hsa-mir-3690-1*** | *CS2FRA* | intron | rs183044496 | C>G |
| ***hsa-mir-3692*** | *ZDHHC14* | intron | rs185691679 | G>A |
| ***hsa-mir-3910-2*** | *ROR2* | intron | rs149611497 | ATGCC>- |
| ***hsa-mir-3939*** | *RP1-167A14.2* | intron | rs76608449 | G>C |
|  |  |  | rs75823810 | C>T |
|  |  |  | rs73024232 | G>A |
| ***hsa-mir-4257*** | *ADAMTSL4* | intron | rs74743733 | G>A |
| ***hsa-mir-4290*** | *RP11-406A20.4* | intron | rs182483446 | G>A |
| ***hsa-mir-4293*** | *FRMD4A* | intron | rs12220909 | G>C |
| ***hsa-mir-4296*** | *CTBP2* | intron | rs182347826 | C>A |
| ***hsa-mir-4301*** | *DRD2* | intron | rs184176277 | G>T |
| ***hsa-mir-4315-1*** | *PLEKHM1* | intron | rs143523766 | C>T |
|  |  | exon | rs147167801 | G>A |
| ***hsa-mir-4461*** | *PCBD2* | intron | rs3928311 | C>T |
| ***hsa-mir-4467*** | *LRWD1* | exon | rs115101071 | G>A |
|  |  | intron | rs76625393 | C>T |
| ***hsa-mir-4517*** | *NFATC2IP* | intron | rs151214931 | T>C |
| ***hsa-mir-4540*** | *PAX5* | intron | rs143699849 | G>A |
|  |  |  | rs60432575 | G>A |
| ***hsa-mir-4641*** | *FOXP4* | intron | rs77674981 | G>A |
| ***hsa-mir-4661*** | *LRRC69* | intron | rs12335005 | G>T |
| ***hsa-mir-4669*** | *RXRA* | intron | rs149603368 | G>A |
| ***hsa-mir-4676*** | *MCU* | intron | rs183259402 | C>T |
| ***hsa-mir-4683*** | *FZD8* | exon | rs151060225 | G>T |
| ***hsa-mir-4691*** | *NDUFS8* | exon, intron | rs181585480 | C>T |
| ***hsa-mir-4695*** | *ALDH4A1 RP13‑279N23.2* | intron | rs79637190 | C>T |
| ***hsa-mir-4706*** | *FNTB* | intron | rs2296320 | C>T |
|  | *CHURC1* | intron | rs72728267 | G>T |
| ***hsa-mir-4707*** | *HAUS4* | exon | rs2273626 | A>C |
|  | *RP11-298I3.5* | intron |  |  |
| ***hsa-mir-4731*** | *PMP22* | intron | rs66507245 | T>A |
| ***hsa-mir-4741*** | *RBBP8* | exon | rs7227168 | C>T |
|  |  |  | rs115479920 | G>A |
| ***hsa-mir-4743*** | *CTIF* | intron | rs141766192 | C>T |
| ***hsa-mir-4747*** | *UHRF1* | intron | rs77046863 | C>T |
| ***hsa-mir-4748*** | *DNM2* | intron | rs76796065 | G>T |
| ***hsa-mir-4756*** | *BCAS1* | intron | rs209426 | A>T |
| ***hsa-mir-4781*** | *TCEANC2* | intron | rs74085143 | G>A |
| ***hsa-mir-4788*** | *HMGB3P13* | exon | rs187884409 | G>A |
| ***hsa-mir-4802*** | *RBM47* | intron | rs112628148 | T>C |
| ***hsa-mir-4804*** | *TNPO1* | intron | rs266435 | C>G |
| ***hsa-mir-5090*** | *LRWD1* | exon, intron | rs3823658 | G>A |
| ***hsa-mir-5197*** | *CTB-57H20.1* | intron | rs77549240 | G>T |
| ***hsa-mir-5585*** | *TMEM39B* | intron | rs141449846 | T>G |
| ***hsa-mir-5589*** | *C3P1* | intron | rs116796353 | A>G |
| ***hsa-mir-6499*** | *FAT2* | intron | rs3734050 | C>T |
| ***hsa-mir-6509*** | *WDR91* | intron | rs145322812 | G>A |
| ***hsa-mir-6717*** | *NDRG2* | exon | rs150596480 | C>T |
|  |  |  | rs117650137 | G>A |
| ***hsa-mir-6721*** | *AGPAT1* | exon | rs187833281 | G>A |
| ***Unvalidated polymorphisms – HUMAN*** | | | | |
| ***hsa-mir-548aq*** | *IGF2BP2* | intron | [rs148741643](http://www.ncbi.nlm.nih.gov/projects/SNP/snp_ref.cgi?rs=rs148741643) | T>C |
| ***hsa-mir-644b*** | *DKC1* | intron | rs112159031 | G>A |
| ***hsa-mir-941-1*** | *DNAJC5* | intron | rs113283070 | G>C |
| ***hsa-mir-1234*** | *CPSF1* | exon, intron | rs141140965 | C>G |
| ***hsa-mir-3118-1*** | *AL583842.3* | intron | rs2779672 | A>C |
| ***hsa-mir-3118-2*** | *BX571672.1 BX571672.4* | intron | rs2779672 | A>C |
| ***hsa-mir-3118-3*** | *BX004987.4* | intron | rs11488501 | C>T |
|  |  |  | rs2779672 | A>C |
| ***hsa-mir-3125*** | *TRIB2* | intron | rs33977954 | ->A |
| ***hsa-mir-3156-2*** | *ANKRD30B* | intron | rs113478966 | T>C |
|  |  |  | rs112428304 | C>T |
| ***hsa-mir-3610*** | *RAD21* | exon | rs112072631 | T>G |
| ***hsa-mir-3618*** | *DGCR8* | exon | rs12159555 | C>G |
| ***hsa-mir-4274*** | *SORCS2* | intron | rs141916455 | ->CAC |
| ***hsa-mir-4525*** | *RAB40B* | intron | rs34711227 | ->C |
| ***hsa-mir-4640*** | *DDR1* | exon, intron | rs150797141 | CT>- |
| ***hsa-mir-4749*** | *PTOV1* | intron | rs148982635 | G>A |
| ***hsa-mir-5089*** | *GOSR2 RP11‑156P1.2* | intron | rs111444930 | G>A |
| ***hsa-mir-5194*** | *FAM49B* | intron | rs35804210 | C>- |
| ***hsa-mir-6515*** | *CALR* | intron | rs75453089 | C>T |
| ***Validated polymorphisms - MOUSE*** | | | | |
| ***mmu-mir-466q*** | *Tnik* | intron | [rs52447291](http://www.ncbi.nlm.nih.gov/projects/SNP/snp_ref.cgi?rs=rs52447291) | C>T |
|  |  |  | rs52368901 | A>G |
| ***mmu-mir-698*** | *Inpp5b* | exon, intron | rs27569360 | G>A |
| ***mmu-mir-717*** | *Gpc3* | intron | rs30372501 | A>G |
| ***mmu-mir-1948*** | *Ttc39c* | intron | rs6363056 | A>G |
| ***mmu-mir-3104*** | *Brsk2* | exon | rs38006026 | G>T |
| ***mmu-mir-6241*** | *Gm4675* | intron | [rs31256256](http://www.ncbi.nlm.nih.gov/projects/SNP/snp_ref.cgi?rs=rs31256256) | G>T |
| ***mmu-mir-6538*** | *Rnf144a* | intron | [rs107783255](http://www.ncbi.nlm.nih.gov/projects/SNP/snp_ref.cgi?rs=rs107783255) | G>A |
| ***Unvalidated polymorphisms - MOUSE*** | | | | |
| ***mmu-mir-3058*** | *Nudt4* | intron | rs45674616 | C>T |
| ***mmu-mir-5625*** | *AC105298.2* | exon | rs6377091 | A>G |
| ***Validated polymorphisms - CHICKEN*** | | | | |
| ***gga-mir-1644*** | *Q6IVU9* | intron | rs14076349 | C>T |
| ***gga-mir-1657*** | *RAB38* | intron | rs14934924 | A>G |
| ***gga-mir-1658*** | *Q5ZI61* | intron | rs16681031 | C>G |
|  |  |  | rs16681032 | C>T |
|  |  |  | rs16681033 | ->G |
