# Supplementary material for: Genome-Wide and Species-Wide In Silico Screening for Intragenic MicroRNAs in Human, Mouse and Chicken
Source: PLoS One. 2013 Jun 6;8(6):e65165. doi: 10.1371/journal.pone.0065165 (PMC3675212; doi:10.1371/journal.pone.0065165)
Supplement: Table S2 — Dysregulation of expression in diseases associated with 27 human miRNA/host gene pairs with cross-species conserved co-location. (DOCX) [file pone.0065165.s008.docx]

**Supporting Table S2: Dysregulation of expression in diseases associated with 27 human miRNA/host gene pairs with cross-species conserved co-location.**

| **miRNA gene** | **host gene** | **dysregulated gene expression in matching diseases (miRNA/host gene)**  **(obtained from Gene Expression Atlas)** |
| --- | --- | --- |
| *hsa-let-7g* | *WDR82* | chronic lymphocytic leukemia (↑/↑), colon carcinoma (↓/↑), squamous cell carcinoma (↓/↓) |
| *hsa-mir-101-2* | *RCL1* | small cell lung carcinoma (↑/↓) |
| *hsa-mir-103a-1* | *PANK3* | small cell lung carcinoma (↑/↑), gastric carcinoma (↑/↓), non-small cell lung carcinoma (↑/↑), squamous cell carcinoma (↓/↓), chronic lymphocytic leukemia (↓/↑↓), colon carcinoma (↓/↑↓) |
| *hsa-mir-107* | *PANK1* | small cell lung carcinoma (↑/↑), non-small cell lung carcinoma (↑/↑), adenocarcinoma (↓/↑), chronic lymphocytic leukemia (↓/↑), colon carcinoma (↓/↑), squamous cell carcinoma (↓/↓) |
| *hsa-mir-126* | *EGFL7* | hepatocellular carcinoma (↑/↑), colon carcinoma (↓/↑), non-small cell lung carcinoma (↑↓/↓), small cell lung carcinoma (↓/↓) |
| *hsa-mir-128-1* | *R3HDM1* | chronic lymphocytic leukemia (↓/↑↓) |
| *hsa-mir-128-2* | *ARPP21* | colon carcinoma (↓/↓) |
| *hsa-mir-1306* | *DGCR8* | acute lymphoblastic leukemia (↑/↓), baseline uninfected (↑/↓), breast cancer (↑/↑↓), colorectal adenocarcinoma (↑/↓), superficial transitional cell carcinoma without surrounding carcinoma in situ lesion (↑/↑), T-cell acute lymphoblastic leukemia (↑/↑↓), small cell lung cancer (↑/↑), colon carcinoma (↑/↑), hepatocellular carcinoma, no satellite nodules (↑/↓), erythromyeloblastoid leukemia (↑/↑), Ewing sarcoma (↑/↑), Huntington's disease (↑/↓), chronic myeloid leukemia (↑/↓), colorectal tumor (↑/↑), dermatitis (↑/↓), monoclonal gammopathy of unknown significance (↑/↓), multiple myeloma (↑/↓), presymptomatic Duchenne muscular dystrophy (↑/↓), prostate carcinoma (↑/↑↓), smoldering myeloma (↑/↓), neuroblastoma - poorly differentiated (↓/↑), anaplastic large cell lymphoma (↓/↓), osteosarcoma (↓/↓), breast tumor, normal like (↓/↓), pretreatment primary acute lymphoblastic leukemia (↓/↓), locally advanced breast carcinoma (↓/↓), colon cancer (↓/↓), germ cell tumor (↓/↓), acute myeloid leukemia (↓/↑↓), brain tumor (↓/↓), breast tumor, luminal (↓/↓), juvenile dermatomyositis (↓/↑), lung adenocarcinoma (↓/↓), ovarian tumor, serous (↓/↑), renal cell carcinoma (↓/↓) |
| *hsa-mir-140* | *WWP2* | chronic lymphocytic leukemia (↑/↑), small cell lung carcinoma (↓/↓), squamous cell carcinoma (↓/↓), adenocarcinoma (↓/↓), colon carcinoma (↓/↑) |
| *hsa-mir-15b* | *SMC4* | chronic lymphocytic leukemia (↑/↓), adenocarcinoma (↓/↑↓), colon carcinoma (↓/↑↓) |
| *hsa-mir-16-2* | *SMC4* | chronic lymphocytic leukemia (↑/↓), adenocarcinoma (↓/↑↓), colon carcinoma (↓/↑↓) |
| *hsa-mir-190a* | *TLN2* | gastric carcinoma (↑/↑), colon carcinoma (↑/↑), chronic lymphocytic leukemia (↓/↓) |
| *hsa-mir-211* | *TRPM1* | acute myeloid leukemia (↑/↑↓), lung adenocarcinoma (↑/↓), no inhalation injury (↑/↑), normospermic (↑/↓), juvenile dermatomyositis (↑/↓), Freidriech's ataxia (↑/↑), pancreatic cancer (↑/↓), T-cell acute lymphoblastic leukemia (↓/↑), teratozoospermia (↓/↑), Parkinson's disease (↓/↓), T acute lymphoblastic leukemia (↓/↓), lung carcinoma (↓/↓), periodontitis (↓/↓), prostate cancer (↓/↑↓), small cell lung carcinoma (↓/↓), tumor (↓/↑↓), breast carcinoma (↓/↓), chronic lymphocytic leukemia (↓/↓) |
| *hsa-mir-218-1* | *SLIT2* | chronic lymphocytic leukemia (↓/↓) |
| *hsa-mir-218-2* | *SLIT3* | benign prostatic hyperplasia (↑/↑) |
| *hsa-mir-23b* | *C9orf3* | colon carcinoma (↓/↑↓), benign prostatic hyperplasia (↑/↑) |
| *hsa-mir-24-1* | *C9orf3* | vulvar intraepithelial neoplasia (↑/↑), Freidriech's ataxia (↑/↑), benign prostatic hyperplasia (↑/↑), leiomyosarcoma (↑/↑↓), type I endometrial adenocarcinoma (↑/↑), uterine fibroid (↑/↑), acute myeloid leukemia (↑↓/↑↓), liposarcoma - dedifferentiated (↓/↓), prostate carcinoma (↓/↑↓), Parkinson's disease (↓/↓), breast carcinoma (↓/↑↓), periodontitis (↓/↑↓), pituitary cancer (↓/↓), type II endometrial adenocarcinoma (↓/↓), colon carcinoma (↑/↑↓), non-small cell lung cancer (↑/↑), adenocarcinoma (↓/↑) |
| *hsa-mir-26a-1* | *CTDSPL* | benign prostatic hyperplasia (↑/↑↓), chronic lymphocytic leukemia (↑/↓), prostate carcinoma (↓/↑↓), colon carcinoma (↓/↑), gastric carcinoma (↓/↑) |
| *hsa-mir-27b* | *C9orf3* | colon carcinoma (↓/↑↓), small cell lung carcinoma (↓/↑), adenocarcinoma (↓/↑) |
| *hsa-mir-301a* | *SKA2* | *data not available* |
| *hsa-mir-30c-1* | *NFYC* | benign prostatic hyperplasia (↑/↑) |
| *hsa-mir-30e* | *NFYC* | *data not available* |
| *hsa-mir-32* | *TMEM245* | squamous cell carcinoma (↓/↑), adenocarcinoma (↓/↑), colon carcinoma (↑↓/↑), gastric carcinoma (↑/↓) |
| *hsa-mir-33a* | *SREBF2* | colon carcinoma (↑/↑), chronic lymphocytic leukemia (↓/↓) |
| *hsa-mir-455* | *COL27A1* | benign prostatic hyperplasia (↑/↑) |
| *hsa-mir-499a* | *MYH7B* | *data not available* |
| *hsa-mir-7-1* | *HNRNPK* | colon carcinoma (↑/↑), Huntington's disease (↑↓/↑↓), prostate carcinoma (↑/↑↓), sarcoma (↑/↑), neuroblastoma - poorly differentiated (↑/↑), X-linked agammaglobulinemia (↑/↑), neuroblastoma (↑/↑), ovarian tumor - endometrioid (↑/↑), common variable immunodeficiency (↑/↑), influenza (↑/↑), lung adenocarcinoma - ebv infection (↑/↑), urinary tract infection (↑/↑), cystic fibrosis (↑/↑), aggressive chronic myelogenous leukemia (↑/↑), chronic myeloid leukemia (↑/↑), hepatitis c (↑/↑), breast adenocarcinoma (↑/↑), placental choriocarcinoma (↑/↑), T-cell acute lymphoblastic leukemia (↑/↑), amyotrophic lateral sclerosis (↑/↑), choriocarcinoma (↑/↑), B-cell lymphoma (↑/↑), Ewing sarcoma (↑/↑), lung adenocarcinoma - gemcitabine treated - gemcitabine resistant (↑/↑), acute lymphoblastic leukemia (↑/↑), acute myeloid leukemia (↑/↑↓), acute quadriplegic myopathy (↑/↑), breast cancer (↑/↑), chronic myelogenous leukemia (↑/↑), chronic myelogenous leukemia - indolent (↑/↑), diffuse large B-cell lymphoma (↑/↑), germ cell tumor (↑/↑), HIV infection (↑/↑), malignant melanoma (↑/↓), precursor T lymphoblastic leukemia (↑/↑), superficial transitional cell carcinoma with surrounding carcinoma *in situ* lesion (↑/↑), juvenile dermatomyositis (↑↓/↑↓), lung adenocarcinoma (↑↓/↓), hereditary spastic paraplegia (↓/↓), glioblastoma (↓/↑↓), colon adenocarcinoma (↓/↓), prostate adenocarcinoma (↓/↓), non-small cell lung cancer (↑↓/↑), AIDS-KS - HIV+: nodular (late) stage (↓/↓), ischemic cardiomyopathy (↓/↓), lipoma (↓/↓), myocardial infarction (↓/↓), calpainopathy (↓/↓), malaria - experimentally infected (↓/↑), presymptomatic Duchenne muscular dystrophy (↓/↓), ischemia (↓/↓), spastic paraplegia (↓/↓), Duchenne muscular dystrophy (↓/↓), Emery-Dreifuss muscular dystrophy (↓/↑↓), bipolar disorder (↓/↓), carcinoma *in situ* lesion (↓/↓), dysferlin mutation (↓/↓), emphysema (↓/↓), facioscapulohumeral muscular dystrophy (↓/↓), metabolic syndrome (↓/↓), mitochondrial disorder (↓/↓), monoclonal gammopathy of unknown significance (↓/↓), multiple myeloma (↓/↑), non-ischemic cardiomyopathy (↓/↓), periodontitis (↓/↓), prostate cancer (↓/↑↓), gastric carcinoma (↑/↑↓), chronic lymphocytic leukemia (↑↓/↓) |

↓ - under-expressed compared to the gene’s overall mean expression level in the study; ↑ - over-expressed compared to the gene’s overall mean expression level in the study.
